# Supplementary material for: The REGγ-Proteasome Regulates Spermatogenesis Partially by P53-PLZF Signaling
Source: Stem Cell Reports. 2019 Aug 8;13(3):559–71. doi: 10.1016/j.stemcr.2019.07.010 (PMC6742627; doi:10.1016/j.stemcr.2019.07.010)

Stem Cell Reports, Volume *13*

Supplemental Information

The REGγ-Proteasome Regulates Spermatogenesis Partially by P53-PLZF Signaling

Xiao Gao, Hui Chen, Jian Liu, Shihui Shen, Qingwei Wang, Tracy M. Clement, Brian J. Deskin, Caiyu Chen, Dengpan Zhao, Lu Wang, Linjie Guo, Xueqing Ma, Bianhong Zhang, Yunfei Xu, Xiaotao Li, and Lei Li

Supplemental Figure Legends

Figure S1. Expression of REGγ In Male Mouse Germ Cells. Related to Figure 1.

(A) Immunofluorescence (IF) staining of REGγ in p7, p10 and 2m mouse testes. (Scale bar, 50μm).

(B) The number of pups in breeding trial experiments.

(C) Photograph of mouse testes.

(D) Quantification of mouse whole weight. Error bars represent SEM.

(E) H&E staining of mouse testes. (Scale bar, 50μm).

Figure S2. Decreased Proportion of Undifferentiated Spermatogonia. Related to Figure 3.

(A) IF staining of PLZF and REGγ in control and REGγ$^{-/-}$ testes at p10. The DNA was stained with DAPI. (Scale bar, 50μm).

(B-C) RT-qPCR analysis of marker gene expression in testes at p7 from REGγ$^{+/+}$ and REGγ$^{-/-}$ mouse testes, with actin as an internal control. (n = 3, ** P < 0.01, *** P < 0.001). Error bars represent SEM.

Figure S3. REGγ$^{-/-}$ testes induced p53 expression and apoptosis while reduce PLZF expression. Related to Figure 5.

(A-B) IF staining analysis of p53, REGγ and PLZF in REGγ$^{+/+}$ and REGγ$^{-/-}$ testes. Upper panel: IF staining at p10 (A) and 2m (B), (Scale bar, 50μm).

(C) Western Blotting analysIs of cleaved PARP, PARP, REGγ and β-actin in adult

REGγ$^{+/+}$ and REGγ$^{-/-}$ male testes treated Cisplatin in different time. β-actin was used as a loading control.

(D) Detection of apoptotic cells and SCP3 double staining in the testes of REGγ$^{-/-}$ mice at 2 months of age. (Scale bar, 50μm).

Figure S4. Genetic attenuation of p53 restores spermatogenesis in REGγ $^{-/-}$ mice. Related to Figure 6. (A) Histological appearances of testes at p10 from p53$^{+/-}$REGγ$^{-/-}$ and p53$^{+/+}$REGγ$^{-/-}$ male mice. (Scale bar, 50μm).

Figure S1

A

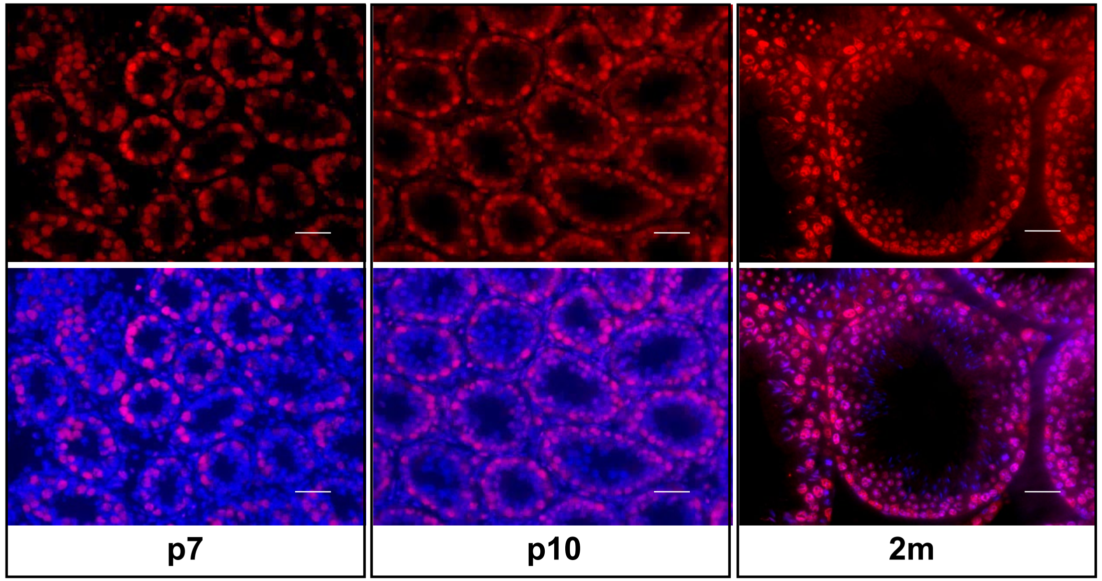

B

The detail information of breeding trials of Figure 1B.

Breeding Cage	Pups (1st litter)	Pups (2nd litter)	Pups (3rd litter)	Pups (4th litter)
REGγ+/+ (♂) x REGγ+/+ (♀)	8	6	9	12
REGγ+/+ (♂) x REGγ+/+ (♀)	5	12	9	6
REGγ+/+ (♂) x REGγ+/+ (♀)	4	9	11	10
REGγ+/+ (♂) x REGγ+/+ (♀)	8	11	9	12
REGγ+/+ (♂) x REGγ+/+ (♀)	7	10	9	13
Total number of pups	180			
Average number of pups per litter	9			
Breeding Cage	Pups (1st litter)	Pups (2nd litter)	Pups (3rd litter)	Pups (4th litter)
REGγ-/-(♂) x REGγ+/+ (♀)	4	5	4	3
REGγ-/-(♂) x REGγ+/+ (♀)	6	6	5	6
REGγ-/-(♂) x REGγ+/+ (♀)	2	4	5	4
REGγ-/-(♂) x REGγ+/+ (♀)	4	8	3	4
REGγ-/-(♂) x REGγ+/+ (♀)	3	6	11	4
Total number of pups	97			
Average number of pups per litter	4.85			

C

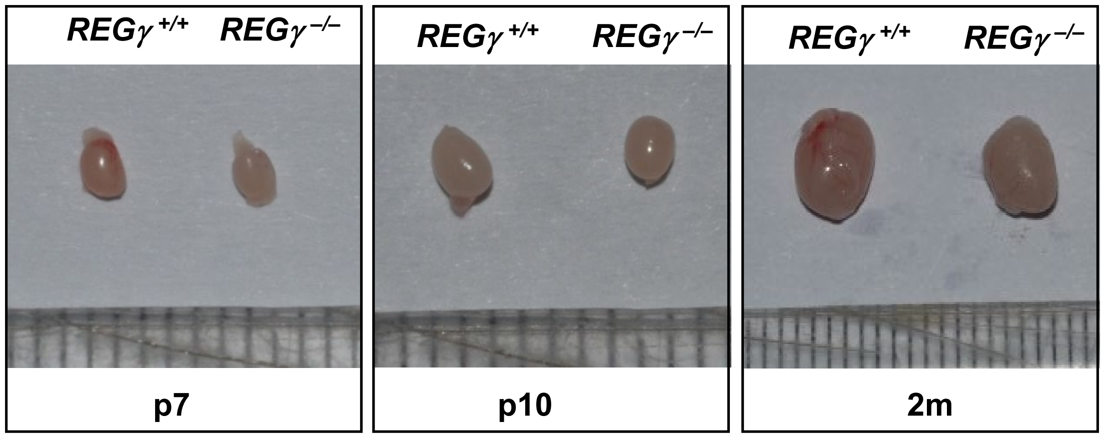

D

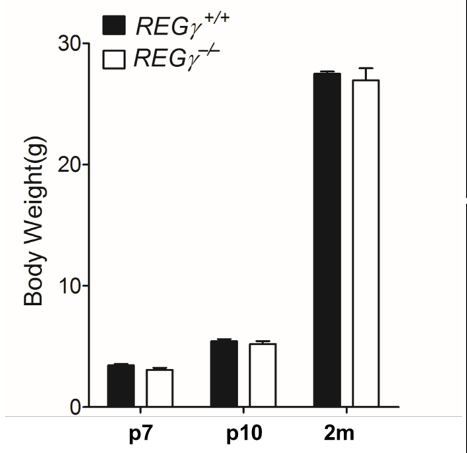

E

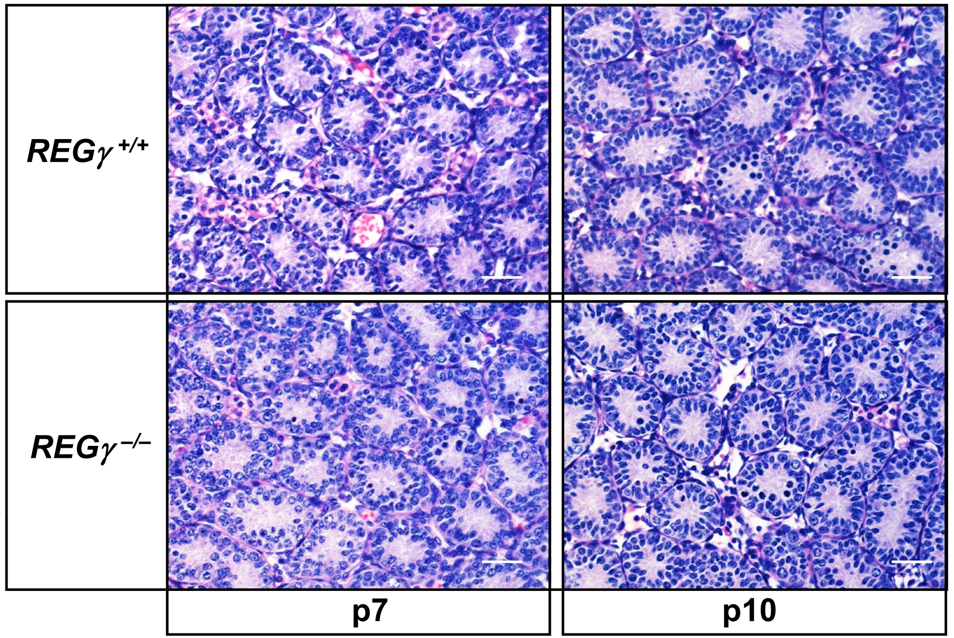

Figure S2

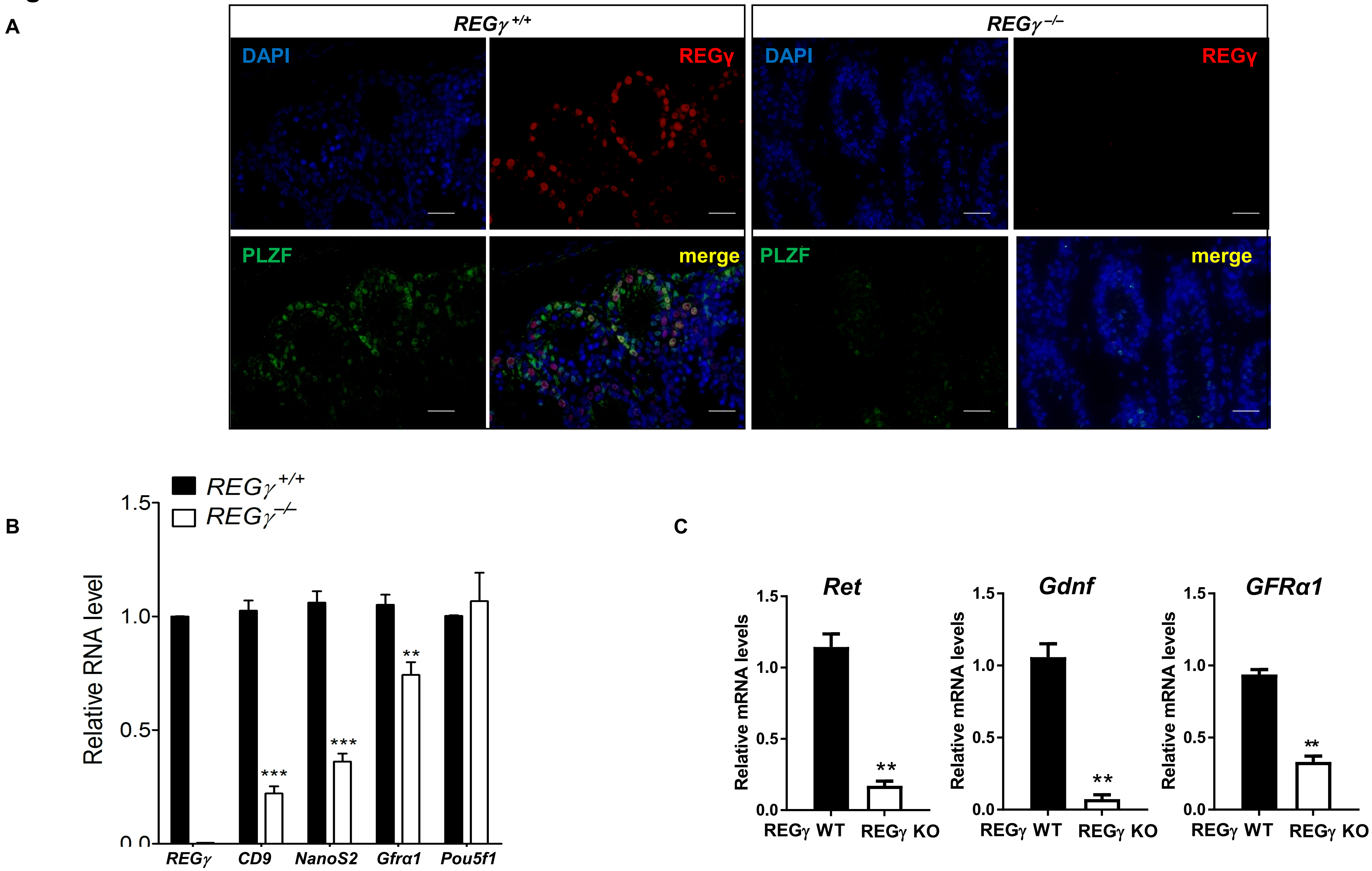
A
REGγ +/+
REGγ −/−
DAPI
REGγ
PLZF
merge
B
REGγ +/+
REGγ −/−
Relative RNA level
1.5
1.0
0.5
0.0
***
***
**
REGγ
CD9
NanoS2
Gfrα1
Pou5f1
C
Ret
Gdnf
GFRα1
Relative mRNA levels
1.5
1.0
0.5
0.0
**
REGγ WT
REGγ KO

Figure S3

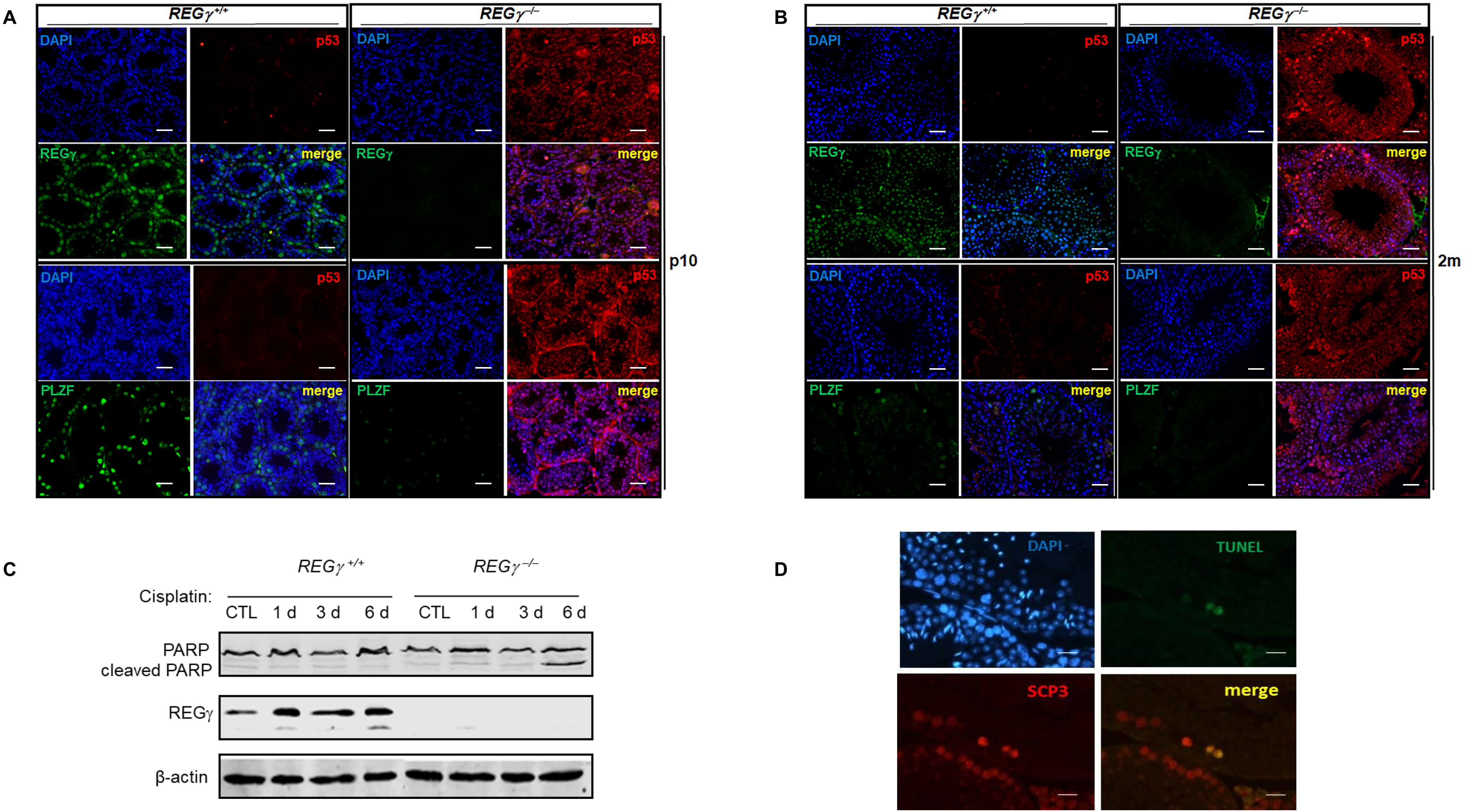
A
REGγ+/+
REGγ-/-
DAPI
p53
REGγ
merge
PLZF
p10
B
2m
C
REGγ +/+
REGγ -/-
Cisplatin:
CTL
1 d
3 d
6 d
PARP
cleaved PARP
REGγ
β-actin
D
DAPI
TUNEL
SCP3
merge

Figure S4

A

p53^+/–^REGγ^–/–^

p53^+/+^REGγ^–/–^

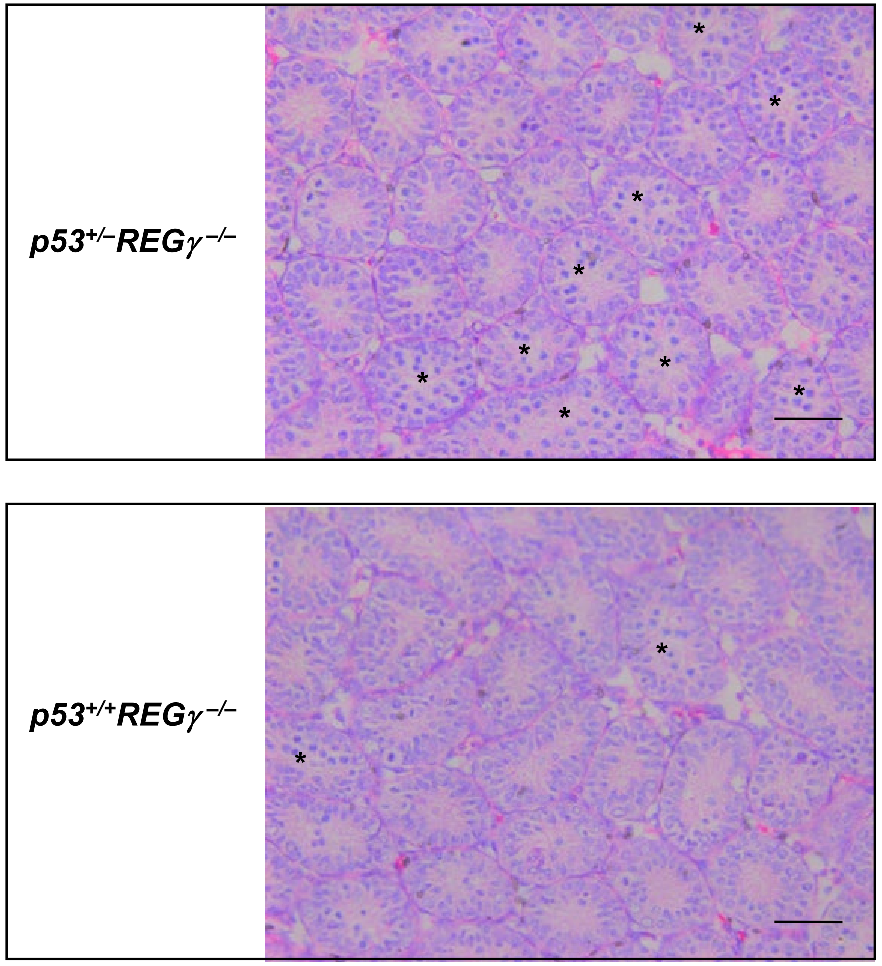

Supplement: Document S1. Figures S1–S4 [file mmc1.pdf]
