# Supplementary material for: The REGγ-Proteasome Regulates Spermatogenesis Partially by P53-PLZF Signaling
Source: Stem Cell Reports. 2019 Aug 8;13(3):559–71. doi: 10.1016/j.stemcr.2019.07.010 (PMC6742627; doi:10.1016/j.stemcr.2019.07.010)

Stem Cell Reports
Article
OPEN ACCESS

The REGγ-Proteasome Regulates Spermatogenesis Partially by P53-PLZF Signaling

Xiao Gao,[1,6] Hui Chen,[1,6] Jian Liu,[2,6,7] Shihui Shen,[1,6] Qingwei Wang,[1] Tracy M. Clement,[3] Brian J. Deskin,[4] Caiyu Chen,[1] Dengpan Zhao,[1] Lu Wang,[1] Linjie Guo,[1] Xueqing Ma,[1] Bianhong Zhang,[1] Yunfei Xu,[5] Xiaotao Li,[2] and Lei Li[1,*]

[1]Shanghai Key Laboratory of Regulatory Biology, Institute of Biomedical Sciences, School of Life Sciences, East China Normal University, 500 Dongchuan Road, Shanghai 200241, China
[2]Department of Molecular and Cellular Biology, Baylor College of Medicine, Houston, TX 77030, USA
[3]Department of Veterinary Physiology and Pharmacology, Texas A&M University, College Station, TX, USA
[4]Epigenetic & Stem Cell Biology Laboratory, National Institute of Environmental Health Sciences, Research Triangle Park, NC 27709, USA
[5]Department of Urology, Shanghai Tenth People's Hospital, Tongji University, Shanghai 200072, China
[6]Co-first author
[7]Present address: Reproductive & Developmental Biology Laboratory, National Institute of Environmental Health Sciences, Research Triangle Park, NC 27709, USA
*Correspondence: lllkzj@163.com
https://doi.org/10.1016/j.stemcr.2019.07.010

SUMMARY

Development of spermatogonia and spermatocytes are the critical steps of spermatogenesis, impacting on male fertility. Investigation of the related regulators benefits the understanding of male reproduction. The proteasome system has been reported to regulate spermatogenesis, but the mechanisms and key contributing factors *in vivo* are poorly explored. Here we found that ablation of REGγ, a proteasome activator, resulted in male subfertility. Analysis of the mouse testes after birth showed there was a decreased number of PLZF^{+} spermatogonia and spermatocytes. Molecular analysis found that REGγ loss significantly increased the abundance of p53 protein in the testis, and directly repressed PLZF transcription in cell lines. Of note, allelic p53 haplodeficiency partially rescued the defects in spermatogenesis observed in REGγ-deficient mice. In summary, our results identify REGγ-p53-PLZF to be a critical pathway that regulates spermatogenesis and establishes a new molecular link between the proteasome system and male reproduction.

INTRODUCTION

Spermatogenesis is a highly complex and organized process of sperm cell development (Walker, 2009). It can broadly be categorized into three stages: the mitotic proliferation of spermatogonia, the meiotic division into haploid germ cells, and spermiogenic differentiation (Zhou et al., 2016). Spermatogonial stem cells (SSCs) are critical for this whole process (Walker, 2009; Wang et al., 2016; Xu et al., 2017; Zhang et al., 2016; Zhou et al., 2016). These SSCs undergo both self-renewal and differentiating divisions and serve as the precursors to spermatozoa (Lovasco et al., 2015). The ability of SSCs to self-renew is restricted to undifferentiated spermatogonia (Takubo et al., 2008). Some transcription factors, such as PLZF, P53, Bcl6b, Lhx1, Etv5, and Id4, have been reported to regulate SSC self-renewal and proliferation (Beumer et al., 1998; Helsel et al., 2017; Kubota et al., 2004; La et al., 2018; Oatley et al., 2006).

PLZF (promyelocytic leukemia zinc finger, also known as *Zfp145*), a transcriptional repressor, regulates the epigenetic state of the undifferentiated spermatogonia and is directly involved in self-renewal and maintenance of the SSC pool (Buaas et al., 2004). PLZF binds to DNA through nine Krüppel-like C2-H2 zinc fingers and an N-terminal BTB/POZ domain in a sequence-specific manner (Li et al., 1997). Overexpression of PLZF induces cell-cycle arrest at the G1 to S transition and represses the expression of pro-proliferative genes, including cyclin A, CCNA2, and MYC (Costoya et al., 2008). PLZF protein inactivates itself in an autologous feedback loop by binding to its promoter, causing rapid exhaustion of the proliferative spermatogonial compartment (Costoya et al., 2004; Filipponi et al., 2007). Mice lacking PLZF show a progressive lack of spermatogonia in the tubules and impaired spermatogenesis, which consequently causes infertility (Buaas et al., 2004; Costoya et al., 2004; Filipponi et al., 2007; Hobbs et al., 2010). Therefore, identification of PLZF regulatory mechanisms will help to understand the SSC fate decisions underlying male fertility.

The p53 family of genes (p53, p63, and p73) are transcription factors and regulate DNA repair, cell-cycle progression, and programmed cell death (Flores et al., 2002; Oren, 1994; Roos and Kaina, 2006). p53 plays an important role in apoptosis during normal spermatogenesis and DNA quality control in spermatocytes (Baum et al., 2005; Beumer et al., 1998; Marcet-Ortega et al., 2017). p53 knockout mice exhibited lower levels of DNA repair during spermatogenesis (Schwartz et al., 1999). Yet hyperactivation of p53 is detrimental to spermatogenesis as well (Fujisawa et al., 2001). Therefore, p53 seems to be a critical

This is an open access article under the CC BY license (http://creativecommons.org/licenses/by/4.0/).

regulator of spermatogenesis; however, the direct mechanisms remain unclear.

REGγ (also known as PA28γ or PSME3) is a member of the 11S proteasome. REGγ binds and activates the 20S proteasome to promote ATP- and ubiquitin-independent protein degradation, a non-typical degradation pathway (Li et al., 2006, 2007). Steroid receptor coactivator-3 was the first identified target of REGγ in this non-typical degradation pathway (Li et al., 2006). Since then, additional targets have been reported, such as the cyclin-dependent kinase inhibitor p21 (Chen et al., 2007; Li et al., 2007), casein kinase (CK) 1δ (Li et al., 2013), SirT1 (Dong et al., 2013), GSK-3β (Chen et al., 2017a, 2017b; Li et al., 2015), hemoglobin (Zuo et al., 2017), and IkBε (Xu et al., 2016). Of note, the degradation of p53, which is essential for spermatogenesis, also can be facilitated by REGγ through MDM2-mediated ubiquitination (Li et al., 2013; Liu et al., 2010; Zhang and Zhang, 2008). Meanwhile, REGγ was found to be expressed in almost all cell types of the mouse testis, including spermatogonial cells, spermatocytes, Leydig cells, Sertoli cells, and spermatid cells (Yu et al., 2010). Interestingly, attenuated proteasome function, such as the knockout of proteasome activator PA200, resulted in defects in spermatogenesis (Khor et al., 2006). However, complete infertility was only observed in double knockout mice in which both REGγ and PA200 were ablated, but not in each single knockout mouse (Huang et al., 2016). These mouse phenotypes and REGγ's regulation of p53 suggest that REGγ may regulate male fertility by the regulation of spermatogenesis.

Herein we report the impact of the proteasome activator, REGγ, for spermatogenesis. We used a mouse model as well as human and mouse cell lines to investigate the underlying mechanism of REGγ deletion in spermatogenesis. We found that REGγ knockout reduced male mouse fertility, with mice exhibiting defects in spermatogenesis. Defects include decreased numbers of PLZF-expressing spermatogonia. This appears to be due to the loss of negative regulation of p53 by REGγ in REGγ null mice. Furthermore, we showed that PLZF expression was negatively regulated by p53 at the transcriptional level in GC-1 cells. Notably, genetic attenuation of p53 partially restores spermatogenesis in REGγ null mice. Taken as a whole, our results identify REGγ as a critical factor required for normal PLZF-expressing spermatogonial stem cell representation and spermatogenesis, partially by regulation of the p53-PLZF pathway.

RESULTS

REGγ Deficiency in Mouse Testes Decreases Sperm Concentration and Activity, Causing Male Subfertility

Previously we showed that REGγ was ubiquitously present in nearly all mouse tissues, with especially high expression in testes (Yu et al., 2010). Here we examined REGγ expression within the mouse testis by conducting immunohistochemical analyses of mouse testes at post-natal days 7 (P7) and 10 (P10) and at 2 months (2m). The P7 stage was chosen for the investigation of development defects preceding meiosis. The P10 stage was included because it is considered the initiation of meiosis. The 2-month stage represents reproductive maturity. REGγ is expressed continuously in mouse testes during testis development (Figures 1A and S1A). Interestingly, while REGγ is expressed in many cell types in testes at 2m, expression at P7 and P10 was selectively detected in the subpopulation of cells near the basement membrane where spermatogonia are enriched (Figure S1A).

To investigate the role of REGγ in male fertility, REGγ$^{+/+}$ or REGγ$^{-/-}$ adult males were bred with REGγ$^{+/+}$ female littermates. Twenty litters per group were counted for analysis of fertility (Figures 1B and S1B). The average number of pups per litter in REGγ$^{-/-}$ male mice was significantly lower than that of the control group. Testes from REGγ$^{-/-}$ male mice were slightly smaller than those from control littermates at different ages (P7, P10, and 2m) (Figures 1C and S1C), whereas the whole-body weight of REGγ knockout mice was similar to that of wild-type mice (Figure S1D). H&E staining was conducted on testes from 2m (Figure 1D) and P7 and P10 (Figure S1E). Pathological phenotypes in REGγ$^{-/-}$ mouse testes observed at 2m (Figure 1D) included an apparent decrease in the number of spermatogonial cells, primary spermatocytes, and spermatids, which was investigated further beginning with assessments of spermatogenic output.

We collected sperm from REGγ$^{+/+}$ or REGγ$^{-/-}$ littermate adults. REGγ$^{-/-}$ mouse sperm concentrations were dramatically lower than the control mice (Figure 1E). Sperm motility was also attenuated in REGγ$^{-/-}$ mice (Figure 1F). When REGγ$^{-/-}$ and REGγ$^{+/+}$ male mice were mated with super-ovulated REGγ$^{+/+}$ female mice, there was a consistent and significant reduction in the number of REGγ$^{-/-}$ fertilized eggs at the two-cell stage (Figures 1G and 1H). Taken together, REGγ loss in mouse testes results in male subfertility, mainly due to the decreased number of sperm and decreased sperm motility.

The Number of Spermatocytes Undergoing Meiosis in REGγ$^{-/-}$ Testis Is Decreased

Because sperm count depends on the successful mitotic, meiotic, and post-meiotic development of the male germ line (Walker, 2009; Wang et al., 2016), we investigated whether specific phases of germ cell development were affected. To investigate whether the reduced sperm in REGγ deficiency was due to decreased spermatocytes undergoing meiosis, we examined the expression of MVH and SCP3 as markers of meiotic spermatocyte

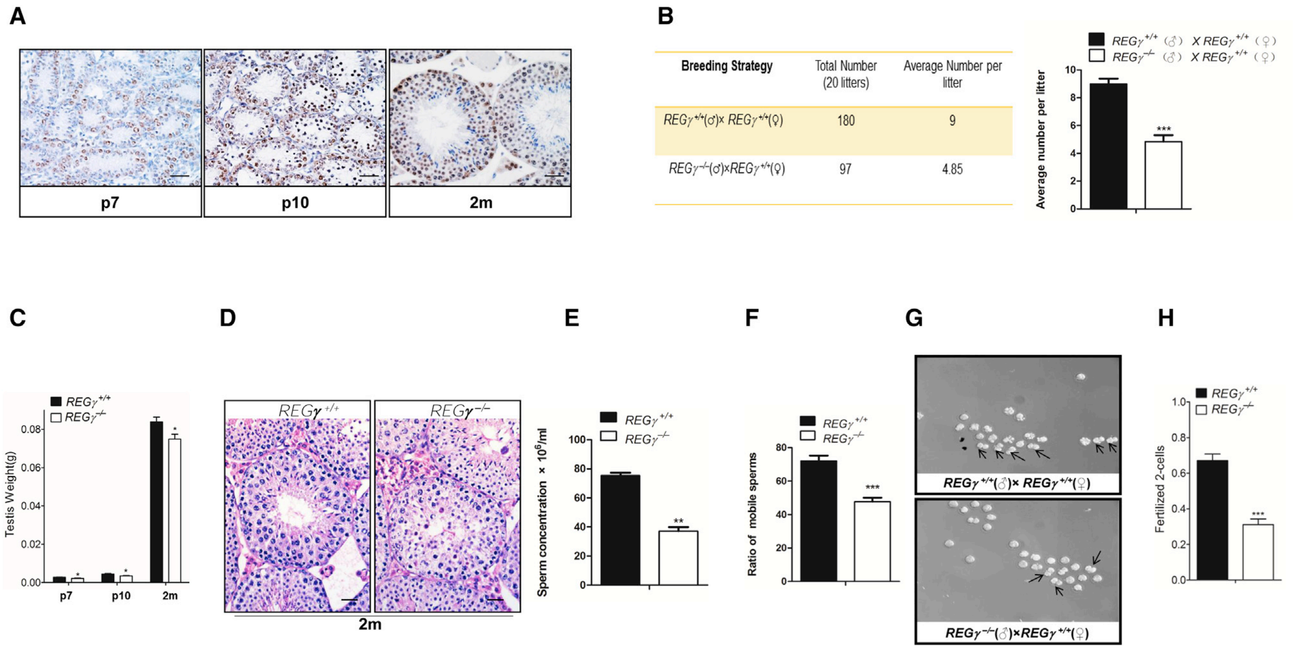

Figure 1. REGγ Deficiency in Mouse Testes Decreases Sperm Concentration and Activity, Causing Male Subfertility

(A) Immunohistochemical (IHC) staining of P7, P10, and 2m mouse testes using REGγ antibodies. Scale bars, 50 μm.

(B) Right panel: 6-month mating studies were performed to assess the fertility in REGγ$^{+/+}$ and REGγ$^{-/-}$ male mice. REGγ$^{+/+}$ (n = 5) or REGγ$^{-/-}$ male mice (n = 5) from littermates were bred with REGγ$^{+/+}$ female mice. The number of pups in each litter was measured from a total of 20 litters in each group (detailed information is listed in Figure S1B). Left panel: the averaged pups per litter per breeding cage was analyzed by paired t test ($p < 0.001$, p values were analyzed by two-tailed t test). *$p < 0.05$, **$p < 0.01$, ***$p < 0.001$. Error bars represent mean ± SEM.

(C) Statistical analysis of testes weight of P7, P10, and adult mice (n = 5, *$p < 0.05$, p values were analyzed by two-way ANOVA). Error bars represent mean ± SEM.

(D) H&E staining of mouse testes at 2 months. Scale bars, 50 μm.

(E) The REGγ$^{-/-}$ and REGγ$^{+/+}$ sperm concentration was measured (n = 10, **$p < 0.01$, p values were analyzed by two-tailed t test). Error bars represent mean ± SEM.

(F) The sperm motility in REGγ$^{-/-}$ and REGγ$^{+/+}$ mice was assessed and is expressed as the percent of total sperm (n = 10, ***$p < 0.001$). Error bars represent mean ± SEM.

(G) Representative examples of two-cell stage fertilization by REGγ$^{-/-}$ and REGγ$^{+/+}$ males.

(H) Quantitation of fertilization rate as observed at the two-cell stage in (D) (n = 5, ***$p < 0.001$, p values were analyzed by two-tailed t test). Error bars represent mean ± SEM.

development. Both immunohistochemical (IHC) and immunofluorescence staining displayed an apparent loss of SCP3^{+} cells in the center of the seminiferous tubules in REGγ$^{-/-}$ mouse testes at P10, indicating a reduction in the number of spermatocytes (Figures 2A–2C). Western blot analyses also showed the reduction of the expression of MVH and SCP3 in REGγ$^{-/-}$ mouse testes at P10 (Figure 2D). Moreover, DNA flow cytometry analysis of DNA content showed that the number of tetraploid primary spermatocytes was decreased in REGγ$^{-/-}$ testes compared with the control testes (Figures 2E and 2F). The expression of germ cell differentiation genes, *Dazl*, *Scp2*, and *Scp3*, was also decreased in REGγ$^{-/-}$ testes (Figure 2G). Collectively these data suggest that the absence of REGγ in mutant mice resulted in the decrease of spermatocytes in the seminiferous epithelium.

REGγ Loss Decreases the Number of PLZF^{+} Spermatogonial Cells

The decrease in spermatocytes undergoing meiosis in REGγ$^{-/-}$ mice suggested potential defects in the early stages of spermatogenesis. To assess the development of spermatogonia, we investigated the expression of PLZF. PLZF is not only a well-known marker specific for undifferentiated spermatogonia in testis but also a critical regulator of germ cell development. In wild-type mouse testes, co-localization of REGγ and PLZF was observed at P7 (Figure 3A) and P10 (Figure S2A), indicating that

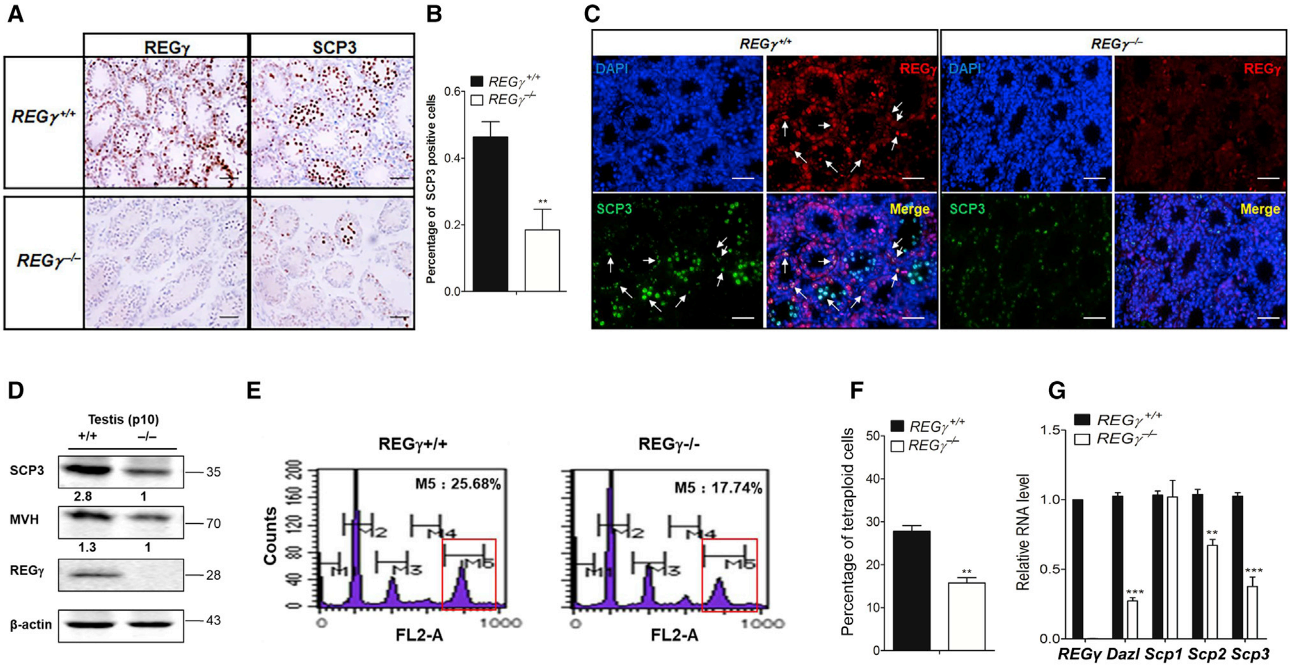

Figure 2. Meiosis in REGγ$^{-/-}$ Testis Is Impaired

(A and B) IHC analysis of meiotic prophase germ cell marker SCP3 and REGγ in REGγ$^{+/+}$ and REGγ$^{-/-}$ testes at P10 (A). Scale bars, 50 μm.
(B) The percent of total cells in (A) that are SCP3^{+}.
(C) Immunofluorescence (IF) staining of REGγ and SCP3 in control and REGγ$^{-/-}$ testes at P10. Scale bars, 50 μm. The white arrows point out the representative cells with the co-localization of REGγ and SCP3.
(D) Western blotting analyses of SCP3, MVH, and REGγ in control and REGγ$^{-/-}$ testes at P10. β-Actin was used as the loading control.
(E and F) Flow cytometry analysis of tetraploid DNA content in adult control and REGγ$^{-/-}$ testes (E) (n = 3 per genotype, $^{**}p < 0.01$, p values were analyzed by two-tailed t test). (F) Quantitative assessment of tetraploid spermatocytes in Figure 3E (n = 3, $^{**}p < 0.01$). Error bars represent mean ± SEM.
(G) Real-time qRT-PCR analysis of marker gene expression in P10 REGγ$^{+/+}$ and REGγ$^{-/-}$ male testes, using actin as the internal control (n = 3, $^{**}p < 0.01$, $^{***}p < 0.001$). Error bars represent SEM (n = 3, $^{**}p < 0.01$, p values were analyzed by two-way ANOVA).

undifferentiated spermatogonia express REGγ. Of note, loss of REGγ resulted in a dramatic decrease of PLZF-expressing spermatogonial cells (Figures 3A–3C). In line with the histological staining results, a drastic reduction of PLZF was seen in whole-testis lysates of P7 REGγ-deficient mice compared with the control (Figure 3D), and *Plzf* transcripts were also reduced (Figure 3E). Considering that we were using a developmental whole-animal knockout model, this decrease might be due to a defect earlier in germ cell development. Therefore, we observed PLZF expression just after birth (P1). The number of PLZF-expressing SSCs was decreased in P1 REGγ-deficient mice compared with control (Figure 3F). At P10, PLZF and SCP3 staining were also reduced (Figure 3G); however, the ratio of SCP3^{+} cells to PLZF^{+} cells in REGγ-deficient testes also indicates a decrease in the abundance of PLZF-expressing cells relative to SCP3-expressing cells compared with the wild-type group (Figures 3G and 3H). This suggests that the decreased number of spermatocytes in REGγ$^{-/-}$ mouse testes is because of fewer PLZF^{+} spermatogonial cells, rather than a defect of meiosis. Furthermore, the expression of spermatogonial development marker genes, including *Cd9*, *Nanos2*, and *Gfrα1*, was significantly lower in the REGγ$^{-/-}$ testes at P7 (Figure S2B). Together, REGγ loss leads to a reduced number of PLZF^{+} spermatogonial cells in the postnatal testis.

REGγ is widely expressed in somatic cells and germ cells by 2m, and SSC development requires somatic niche factors including glial cell-derived neurotrophic factor (GDNF), which is produced by Sertoli cells, and signals through the SSC cell surface receptors RET and GFRα1 (Hofmann, 2008). Therefore, the effect of REGγ knockout on the expression of spermatogonial self-renewal factors that mediate GDNF signaling was examined. Each self-renewal factor tested was downregulated after knockout of REGγ (Figure S2C). Collectively, this indicates that the reduced SSC population in adult REGγ knockout testes could result from non-cell-autonomous mechanisms

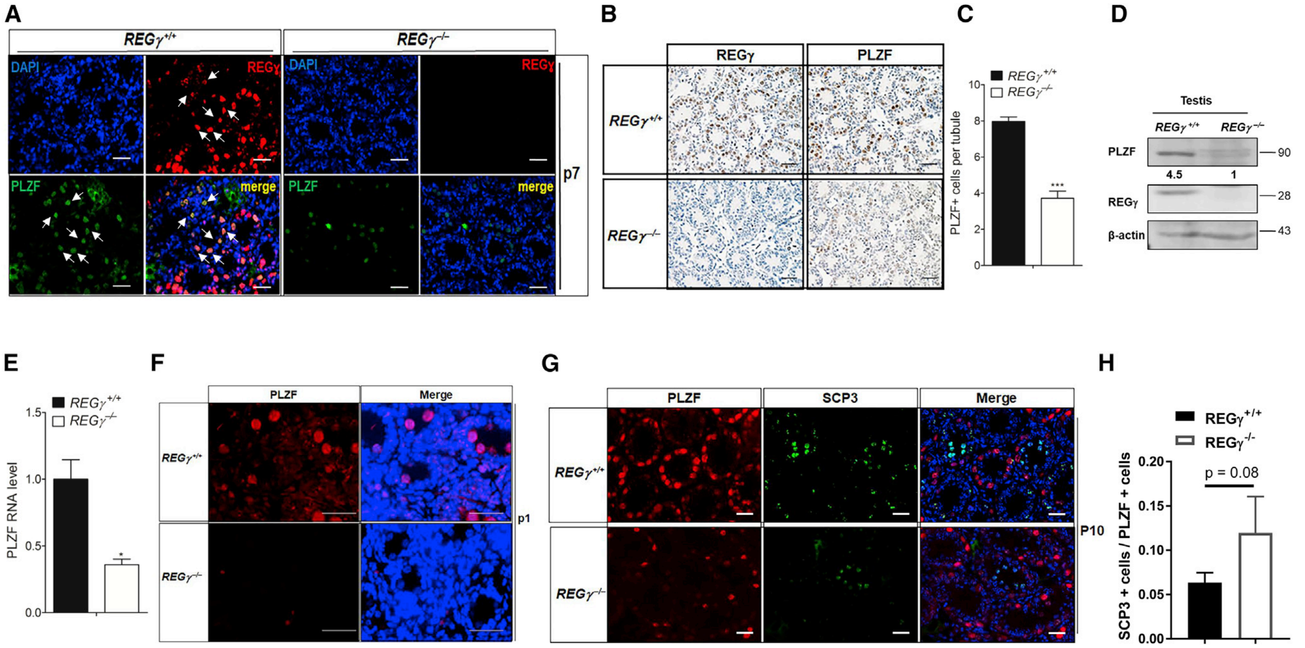

Figure 3. REGγ Loss Decreases the Number of PLZF $^{+}$ Spermatogonial Cells and SSCs

(A) IF staining of REGγ and the undifferentiated spermatogonia marker PLZF in control and REGγ$^{-/-}$ testes at P7. DNA was stained with DAPI. Scale bars, 50 μm. The white arrows point out the representative cells with the co-localization of REGγ and PLZF.

(B and C) IHC analysis of PLZF and REGγ in REGγ$^{+/+}$ and REGγ$^{-/-}$ testes at P7 (B). Magnification, ×40. Scale bars, 50 μm. (C) Quantification of PLZF^{+} cells per tubule. Twenty tubules were counted and quantified from 4 different mice (n = 4 per genotype, ***$p < 0.001$, p values were analyzed by two-tailed t test). Error bars represent mean ± SEM.

(D and E) Expression of REGγ and PLZF in REGγ$^{+/+}$ and REGγ$^{-/-}$ testes at P7 by western blotting (D) and qRT-PCR (E). β-Actin was used as the loading control. Error bars represent mean ± SEM.

(F) IF staining of PLZF in control and REGγ$^{-/-}$ testes at P1. DNA was stained with DAPI. Scale bars, 50 μm.

(G and H) IF staining of PLZF and SCP3 in control and REGγ$^{-/-}$ testes at P10 (G). DNA was stained with DAPI (blue). Scale bars, 50 μm. (H) Quantification of the ratio of SCP3^{+} between PLZF^{+} cells per view (three views of 40× magnification per mouse and three mice per group; p values were analyzed by two-tailed t test; Error bars represent mean ± SD).

(e.g., GDNF) in addition to disruption of cell-autonomous mechanisms.

P53 Binds to the PLZF Promoter and Negatively Regulates PLZF

The reduced PLZF mRNA expression in REGγ$^{-/-}$ mouse testes (Figure 3E) suggested a potentially transcriptional regulation of PLZF even though there was a decrease of PLZF^{+} cells in whole REGγ$^{-/-}$ testes. Gene sequence analysis identified that the 5′ UTR of mouse *Plzf* gene contains putative p53 DNA binding sites, identical to the consensus p53 binding element (el-Deiry et al., 1992; Menendez et al., 2009) (Figure 4A). Considering that p53 is a well-proven target of REGγ (Ali et al., 2013; Li et al., 2013; Liu et al., 2010), and that p53 plays an essential role in spermatogenesis (Fujisawa et al., 2001), we investigated potential p53-dependent regulation of *Plzf*. We transiently knocked down p53 in the C18-4 cell line (an SSC-derived mouse cell line). Of note, silencing p53 dramatically increased the intracellular mRNA level of *Plzf* (Figure 4B). We then generated a luciferase reporter driven by the *Plzf* promoter and tested the effect of p53 on *Plzf*-luciferase reporter expression in a p53-null cell line (H1299) with transfection of p53 or empty vector. As expected, expression of p53 drastically inhibited *Plzf*-luciferase activity (Figure 4C). We observed dose-dependent repression of *Plzf*-luciferase activity in response to p53 titration via transient transfection of H1299 cells, further confirming p53-mediated repression of *Plzf* (Figure 4D). Of note, this repression was abolished by the deletion of the -583 to -556 p53 response element within the *Plzf* promoter expressed in GC-1 spermatogonial-derived cells (Figure 4E). In response to Nutlin-3 (which acts as an inhibitor of the negative regulation of p53, leading to increased p53 activity), inhibition of the *Plzf* transcript was observed in A549 cells, which express wild-type p53 (Figure 4F). Chromatin immunoprecipitation (ChIP) assays showed that p53 bound to the *Plzf* proximal promoter in A549 cells on Nutlin-3 treatment

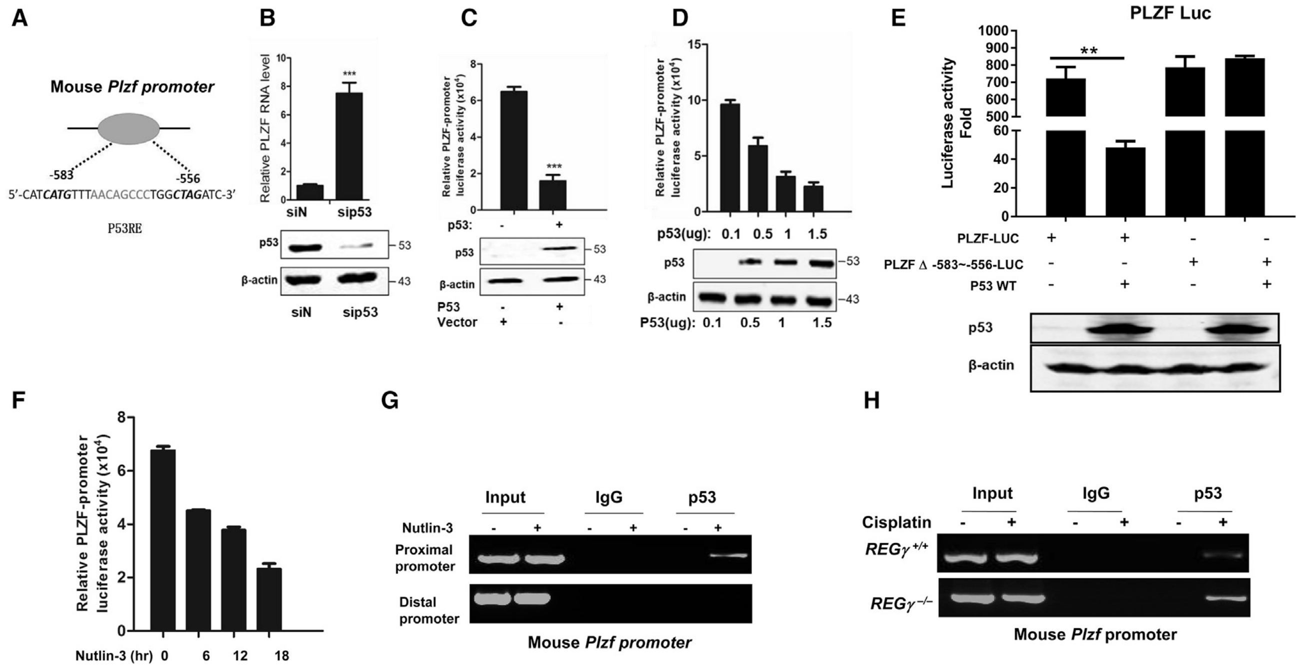

Figure 4. P53 Binds to the *Plzf* Promoter and Negatively Regulates PLZF

(A) Schematic representation of putative p53-responsive elements (p53REs) in the region of the *Plzf* promoter.

(B) Real-time RT-PCR analysis of *Plzf* with transient knockdown of p53 in the C18-4 cell line. Data were obtained from three independent experiments (***$p < 0.001$). Error bars represent SEM.

(C) Analysis of *Plzf*-luciferase reporter activity in the presence of p53 or vector in H1299 cells. Data were obtained from three independent experiments (***$p < 0.001$). Error bars represent SEM.

(D) Analysis of *Plzf*-luciferase reporter activity in a serial concentration of p53 plasmid transfection of H1299 cells. Error bars represent SEM.

(E) Luciferase reporter analysis of the effect of p53 on the wild-type or mutant *Plzf* promoter activity in GC-1 cells by transfection of the plasmids of *Plzf* promoters and p53. Error bars represent SEM.

(F) Analysis of the effect of Nutlin-3 treatment on *Plzf*-luciferase reporter activity in A549 cell lines. Error bars represent SEM.

(G) Chromatin immunoprecipitation (ChIP) assay of the p53 binding on the *Plzf* promoter in A549 cell lines. A549 cells were transfected with *Plzf* proximal and distal promoters. Nutlin-3 treatment is to activate endogenous p53 expression.

(H) ChIP assay of the p53 binding on the *Plzf* promoter in adult REG$\gamma^{+/+}$ and REG$\gamma^{-/-}$ mouse testes with or without 10 mg/kg cisplatin treatment for 24 h.

(Figure 4G). To address whether p53 directly binds to the *Plzf* promoter *in vivo*, we performed ChIP assays using testes from cisplatin-treated REG$\gamma^{+/+}$ and REG$\gamma^{-/-}$ littermates, and cisplatin was used to induce p53 expression. ChIP analysis indicated that p53 was recruited to the *Plzf* promoter region in both REG$\gamma^{+/+}$ and REG$\gamma^{-/-}$ testes (Figure 4H). Taken together, p53 inhibits PLZF at the transcriptional level by directly binding to the *Plzf* promoter.

Elevated p53 Is Associated with Spermatogonial Apoptosis in REG$\gamma^{-/-}$ Testes

Given our finding that p53 regulates *Plzf*, we continued to examine p53 protein expression during spermatogenesis. We speculated that p53 expression would be induced in mouse testes in the context of REGγ knockout based on our previous observations that REGγ promoted the degradation of p53 (Ali et al., 2013; Li et al., 2013; Liu et al., 2010). As expected, a substantial increase of p53 was observed in REG$\gamma^{-/-}$ testes at P7 (Figure 5A), P10 (Figure S3A), and 2m (Figure S3B) stages, while a decrease of REGγ and PLZF was found. To determine whether REGγ downregulates p53 protein levels in spermatogonia via proteasome degradation, overexpression of REGγ in the presence of proteasome inhibition (e.g., MG132) in spermatogonia-derived GC1 cells was conducted (Figure 5B). REGγ overexpression reduced the expression of p53, whereas this reduction was inhibited by MG132 treatment. Meanwhile, MG132 treatment increased p53 protein expression regardless of REGγ or vector transfection, confirming the proteasome regulation of p53 expression in GC-1 cells. In contrast to the decrease of PLZF^{+} cells at birth in REG$\gamma^{-/-}$ mouse testes (Figure 3F), P53 expression was induced (Figure 5C). This raised the

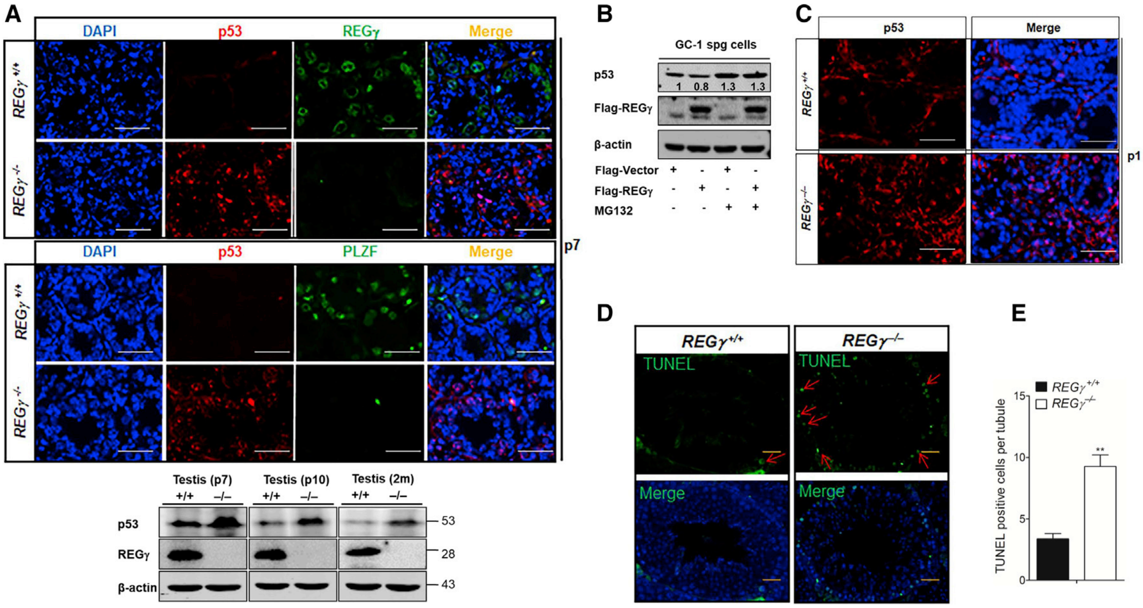

Figure 5. Elevated p53 Is Associated with Spermatogonial Apoptosis in REGγ$^{-/-}$ Testes

(A) Analysis of p53, REGγ, and PLZF in REGγ$^{+/+}$ and REGγ$^{-/-}$ testes. Upper panel: IF staining at P7. Scale bars, 50 μm. Lower panel: western blotting analyses of p53 and REGγ in REGγ$^{+/+}$ or REGγ$^{-/-}$ mouse testes. β-Actin was used as a loading control.

(B) Western blot analysis of protein expression in GC-1 cells after transfection of plasmids of vector and REGγ. Proteasome inhibitor MG132 (10 μM) was used for the 6-h treatment.

(C) Analysis of p53 in REGγ$^{+/+}$ and REGγ$^{-/-}$ testes. Blue is DAPI. Scale bars, 50 μm.

(D and E) Detection of apoptotic cells in the testes of REGγ$^{+/+}$ and REGγ$^{-/-}$ mice at 2 months of age by TUNEL assays (D). Scale bars, 50 μm. (E) The average number of TUNEL^{+} cells in Figure 5F (n = 3, **p < 0.01, p values were analyzed by two-tailed t test).

notion that accumulated p53 expression is a major contributor to the REGγ-loss-induced developmental defect (e.g., loss of PLZF^{+} cells and expression).

Increased p53 can induce cell apoptosis (Fridman and Lowe, 2003). To investigate the possibility of whether elevated p53 induced apoptosis of germ cells in REGγ$^{-/-}$ testes, TUNEL assays were performed on adult REGγ$^{+/+}$ and REGγ$^{-/-}$ testes. As expected, the number of TUNEL^{+} cells was increased in adult REGγ$^{-/-}$ testes compared with control testes (Figures 5D and 5E). In addition, REGγ depletion sensitized testes to cisplatin (an anti-cancer drug)-induced apoptosis, as demonstrated by the accumulation of a cleavage fragment of poly ADP-ribose polymerase (Figure S3C). Interestingly, the majority of TUNEL^{+} cells were spermatocyte-like based on their localization near the base membrane and co-localization with a subset of SCP3^{+} cells (Figure S3D). Our data indicate that the molecular basis for REGγ actions on spermatogenesis is mediated through regulation of p53 in testes.

Genetic Attenuation of p53 Partially Restores Spermatogenesis in REGγ$^{-/-}$ Mice

Because the induced p53 expression downstream of REGγ deficiency has profound effects on spermatogenesis, including attenuated PLZF levels, we hypothesized that decreasing p53 level would partially rescue the defects in REGγ$^{-/-}$ mouse spermatogenesis. We generated combined p53 heterozygous and REGγ-deficient mice (p53$^{+/-}$/REGγ$^{-/-}$) by using p53$^{-/-}$ mice. The p53$^{+/-}$/REGγ$^{-/-}$ mouse spermatogenic phenotypes were analyzed by comparing them with p53$^{+/+}$/REGγ$^{-/-}$. Expectedly, p53 protein expression in p53$^{+/-}$REGγ$^{-/-}$ testes was lower than that of p53$^{+/+}$REGγ$^{-/-}$ testes at P1 (Figure 6A). We next examined the later stages and effects, such as PLZF staining and fertility. Importantly, allelic p53 haplodeficiency led to an increase of PLZF expression at P1 (Figure 6B) and P10 (Figure 6C), as well as the increase of SCP3^{+} cells (Figure 6D). The increase in SCP3^{+} spermatocytes was likely due to the rescue of PLZF^{+} cells in p53$^{+/-}$REGγ$^{-/-}$ testes, not because of increased meiotic entry. This was suggested by the ratio between SCP3^{+} cells and PLZF^{+} cells in p53$^{+/-}$REGγ$^{-/-}$ testes, which is slightly lower than p53$^{+/+}$REGγ$^{-/-}$ testes (Figure 6E). Western blot analysis of whole testes also confirmed the increase of PZLF, and of p21 (a known p53 target) (Kachnic et al., 1999), in p53 haplodeficient mouse testes (Figure 6F). Similarly, the percent of primary spermatocytes was increased based on histomorphology (Figures 6G

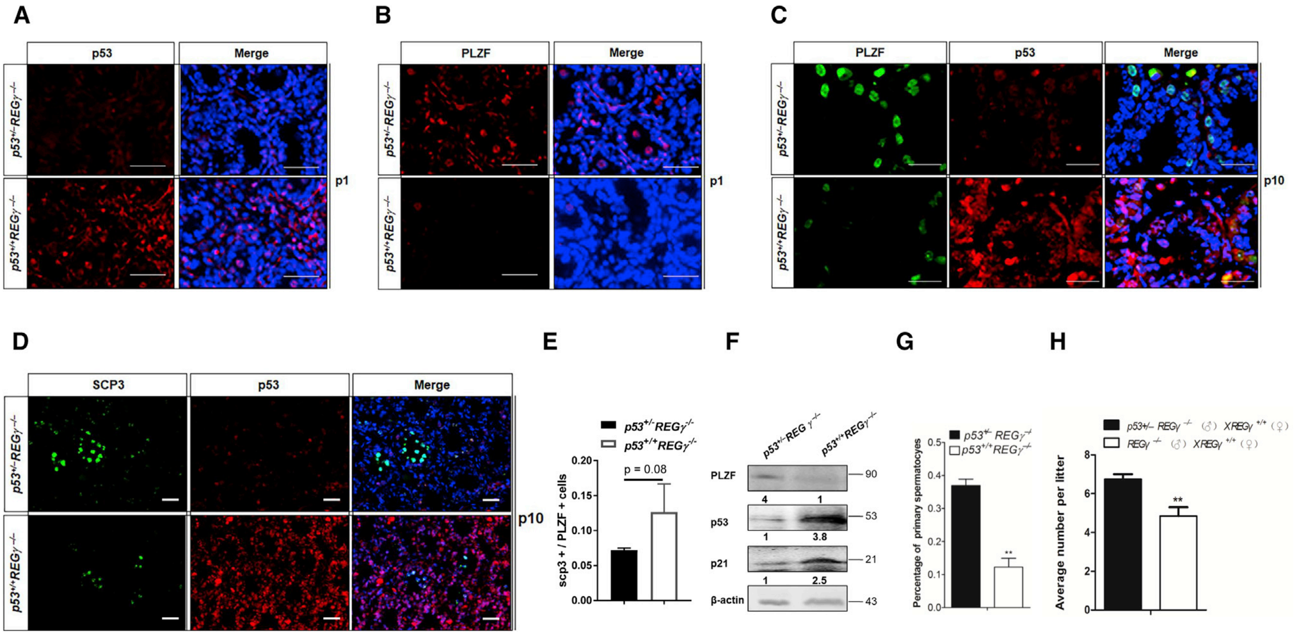

Figure 6. Genetic Attenuation of p53 Partially Restores Spermatogenesis in REGγ$^{-/-}$ Mice
(A and B) IF staining of P53 (A) and PLZF (B) in p53$^{+/-}$/REGγ$^{-/-}$ and p53$^{+/+}$/REGγ$^{-/-}$ testes at P1. Scale bars, 50 μm.
(C–E) IF staining of P53 with PLZF (C) or SCP3 (D) in p53$^{+/-}$/REGγ$^{-/-}$ and p53$^{+/+}$/REGγ$^{-/-}$ testes at P10. Scale bars, 50 μm. (E) The quantification results of (C and D) (three views of 40× magnification per mouse and three mice per group; p53$^{+/-}$/REGγ$^{-/-}$ and p53$^{+/+}$/REGγ$^{-/-}$ are littermates from the breeding cage (p53$^{+/-}$/REGγ$^{-/-}$ × p53$^{+/-}$/REGγ$^{-/-}$). The p values were analyzed by two-tailed t test; error bars represent mean ± SD.
(F) Western blotting analyses of PLZF, p53, and p21 in adult p53$^{+/-}$/REGγ$^{-/-}$ and p53$^{+/+}$/REGγ$^{-/-}$ testes. β-Actin was used as a loading control.
(G) The percent of primary spermatocytes in p53$^{+/-}$REGγ$^{-/-}$ and p53$^{+/+}$REGγ$^{-/-}$ at P10.
(H) p53$^{+/-}$REGγ$^{-/-}$ or p53$^{+/+}$REGγ$^{-/-}$ male mice from littermates were bred with REGγ$^{+/+}$ female mice and the litter size was analyzed (n = 5, $^{**}p < 0.01$, p values were analyzed by two-tailed t test).

and S4). Notably, the average number of pups per litter in p53$^{+/-}$/REGγ$^{-/-}$ male mice was significantly higher than the control group by counting a total of 20 litters in each group (Figure 6H). Based on these observations, we conclude that REGγ regulates spermatogenesis by modulating the p53-PLZF pathway in testes.

DISCUSSION

REGγ has been reported to play important roles in multiple biological processes. However, its function in spermatogenesis is poorly understood. In this study, we reported that REGγ deficiency leads to defects in spermatogenesis and male subfertility. Mechanistically, REGγ appears to be required for the normal development of spermatogenesis by maintaining PLZF^{+} SSCs via suppression of p53, which negatively regulates *PLZF* transcription (Figure 7). Our *in vivo* experiments showed that allelic p53 haplodeficiency in REGγ-deficient mice partially rescued the spermatogenic defects in REGγ$^{-/-}$ mice. Therefore, our study establishes REGγ-p53-PLZF as a new pathway regulating spermatogenesis.

Our current results showed that a developmental defect in spermatogonia may be a major cause of attenuated spermatogenesis in REGγ$^{-/-}$ mice. It is important to note that we also observed that typical spermatogonial self-renewal factors (e.g., *Gdnf*, *Ret*, and *Gfrα1* in Figure S2C) were downregulated after knockout of REGγ. These results suggest that REGγ regulates spermatogenesis through different pathways. The working model (Figure 7) will require testing in spermatogonia, because it remains uncertain how well the SV40-transformed spermatogonial GC1 cell line models spermatogonia. Because REGγ was widely expressed in the adult testis and other tissues, the role of REGγ in specific cells should be further investigated in the future. For example, crossing REGγ floxed mice with Nanos3-Cre mice or Dhh-Cre mice could be used for the investigation of germ cells or Sertoli cells, respectively, and potential disruptions to the

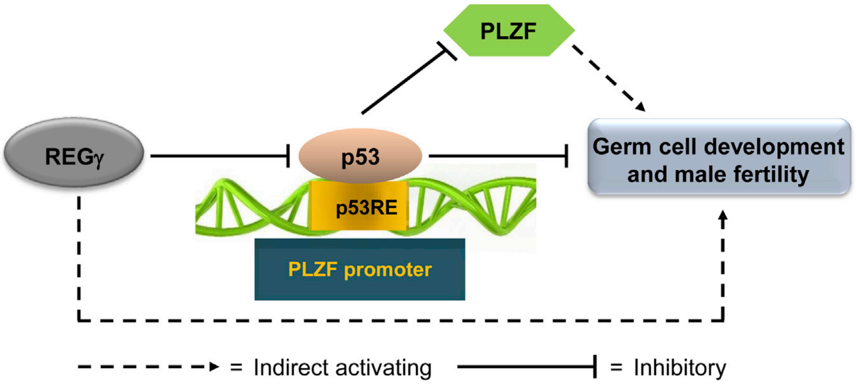

Figure 7. Working Model for the Role of REGγ in Spermatogenesis

REGγ suppresses p53 through regulation of proteasomal degradation. In the absence of p53, negative regulation of the *Plzf* promoter by p53 is also absent. *Plzf* is transcribed and PLZF can function in germ cell development. REGγ deficiency leads to the defect of germ cell development and male subfertility. Mechanistically, REGγ loss results in the accumulation of p53 protein by disruption of REGγ-mediated p53 protein degradation; this subsequently leads to the decreased expression of PLZF through p53 direct inhibition at the transcriptional level. The REGγ-p53-PLZF regulatory pathway provides a new mechanism to understand the role of REGγ in the regulation of spermatogenesis.

hypothalamic-pituitary axis in REGγ$^{-/-}$ mice should be considered in future studies in addition to direct effects on testicular function and SSCs (Matson et al., 2011; Matsumoto and Bremner, 1987).

PLZF is an intrinsic factor, whose loss causes the progressive failure of SSC development in spermatogenesis (Buaas et al., 2004). In our data, male mice lacking REGγ undergo progressive testis atrophy and infertility with age, which is reminiscent of the testis phenotype in PLZF$^{-/-}$ mice. The loss of PLZF in differentiating spermatogonia observed in this study could suggest an imbalance in SSC fate decisions in REGγ$^{-/-}$ mice consistent with the PLZF$^{-/-}$ mouse phenotype. This is supported by the decrease, in REGγ$^{-/-}$ mice, of GDNF, Ret, and Gfra1, which are required for SSC development, and is also consistent with our previous finding that proteasome activities were decreased more dramatically in older REGγ-deficient mice (Lv et al., 2016). Considering that SSC maintenance is regulated by various factors other than PLZF, such as GDNF (Meng et al., 2000), TAF4b, and Ngn3 (Buaas et al., 2004; Costoya et al., 2004; Falender et al., 2005), other mechanisms of REGγ loss leading to the decreased SSCs cannot be excluded.

The tumor suppressor p53 has been implicated in the regulation of SSC proliferation and spermatogonial differentiation (Chen et al., 2012; Marcet-Ortega et al., 2017). In our current study, we uncover a new role for p53 function in spermatogenesis where p53 functions to transcriptionally repress PLZF by directly binding to the *Plzf* promoter. Interestingly, Choi et al. (2014) reported that PLZF repressed transcription of TP53 and also reduced p53 protein stability by ubiquitination. This indicates a regulatory loop between p53 and PLZF. In line with p53's role in cell apoptosis, we found that REGγ knockout mice had more apoptotic cells in testes. Interestingly, the majority of TUNEL^{+} cells were SCP3^{+} cells localized near the basement membrane. It is not clear if this indicates a delay in apoptosis until the meiotic prophase or the precocious expression of SCP-3, as has been seen in SSCs in other mutants. It is necessary to investigate which p53 downstream gene or pathway plays a critical role in the regulation of the apoptosis of spermatogonia and/or spermatocytes in future studies.

In summary, genetic ablation of REGγ in mice leads to the accumulated p53 protein, decreased PLZF expression and PLZF^{+} SSCs, and germ cell defects, eventually causing male subfertility (Figure 7). Therefore, our study identifies REGγ as a key player in the regulation of SSCs and spermatogenesis, and deepens our understanding of the proteasome system in the regulation of reproduction. The clinical application of our findings, such as targeting the REGγ-p53-PLZF pathway in human azoospermia, deserves further investigation and represents an attractive direction.

EXPERIMENTAL PROCEDURES

Mice Maintenance

REGγ$^{-/-}$ mice with C57BL/6 genetic background were acquired from John J. Monaco (University of Cincinnati College of Medicine, Cincinnati, OH) (Barton et al., 2004). P53$^{+/-}$ C57BL/6 mice were purchased from the Model Animal Research Center of Nanjing University. Mice were bred in the Animal Core Facility by following procedures approved by the Institutional Animal Care and Use Committee of East China Normal University.

Measurement of Mouse Sperm Concentration and Sperm Motility

Epididymides from 8- to 10-week-old mice were placed in 500 μL pre-warmed sperm Preparation Medium (Origio, Måløv, Denmark) in a concave glass dish placed in a 37°C water bath for 5 min to fully release the sperm. Tissue fragments were removed, and the sperm-containing culture solution was aspirated using a very fine glass siphon and placed into an HTM-IVOS sperm viability meter. When the sperm concentration was high, samples were further diluted with the medium before performing the measurement using the default program of the machine.

Male Fertility and Fertilization Competency

Continuous mating studies were performed to assess male fertility. Five male mice from each genotype of 8–10 weeks of age were mated to wild-type C57BL/6J females until each produced four litters. The number of pups in each litter was recorded for a total of 20 litters in each group. To further assess fertilization competency in REGγ knockout males, we assessed fertilization competence by observing fertilization rates the day after the mating. Ten 8- to 10-week-old wild-type C57BL/6J female mice were injected with 5 U of pregnant mare serum gonadotropin per mouse. After 24 h, each mouse was injected with 5 U of human chorionic gonadotropin. After 8 h, they were mated with wild-type or REGγ knockout males (five pairs for each genotype) overnight. Female mice were sacrificed by cervical dislocation the next morning. The oocytes and zygotes were collected and placed in human tubal fluid medium in a CO_2 cell culture incubator for 24 to 48 h. The proportion of fertilized eggs in the two-cell stage was then counted and statistical analysis (t test) was performed.

Antibodies, Cell Lines, and Transfections

The following primary antibodies were used: REGγ antibody (Invitrogen, catalog no. 38–3900), p21 antibody (BD Pharmingen, catalog no. 556430), β-actin antibody (Sigma, catalog no. A5316), PLZF antibody (Santa Cruz, catalog no. sc-28319), p53 antibody (Novocastra Laboratories, NCL-p53-CM5p), SCP3 antibody (Abcam, catalog no. ab15093), MVH antibody (Abcam, catalog no. ab13840). C18-4 is a spermatogonial stem cell line with wild-type p53. H1299 is a lung cancer cell line without endogenous p53. A459 is a lung cancer cell line with wild-type p53. GC-1 is a spermatogonial-derived cell line. All the cells are from ATCC and cultured following ATCC standard protocols. The plasmids were transfected into cells using Lipofectamine 2000 (Invitrogen), and the construction of these plasmids is described in the related Experimental Procedures. Cell lysates were collected for protein examination 2 days after transfection. MG132 (10 μM) or Nutlin-3 (10 μM) was used to treat cells for 6 h, and 10 mg/kg of cisplatin was used to treat the mice for 24 h before the sacrifice.

DNA Flow Cytometry Analysis

Testes obtained from 8- to 10-week-old male mice were washed three times aseptically in DMEM. Sertoli cells were isolated from mouse testis biopsies using the two-step enzymatic digestion as follows. First, seminiferous tubules were obtained after being treated with an enzymatic solution containing collagenase type IV (2 mg/mL) and DNAse I (10 μg/mL) in DMEM/F12 at 37°C for 10 min. After washing to remove interstitial cells, Sertoli cells were obtained using second enzymatic digestion with 4 mg/mL collagenase IV, 2.5 mg/mL hyaluronidase (Sigma), 2 mg/mL trypsin (Sigma), and 1 μg/μL DNAse I, followed by differential plating. In brief, cell suspensions were seeded into culture plates in DMEM/F12 (Gibco) supplemented with 10% FBS and incubated at 34°C in 5% CO_2 for 3 h. Flow cytometry analysis of DNA content was conducted as described previously (Zhu and Gooderham, 2002). In brief, cellular DNA content was determined by propidium iodide-staining flow cytometry. Seventy percent ethanol was used to fix cells at −20°C for 1 h. Cells were then resuspended to 1 mL using PBS containing propidium iodide (5 μg/mL) and RNase A (0.1 mg/mL). The suspensions were incubated at 37°C for 30 min. The ploidy determination of nuclei was estimated by flow cytometry DNA content.

RNAi and RNA Analyses

ON-TARGETplus TP53 smart pool small interfering RNAs (siRNAs) (L-003329-00-0005, Dharmacon) and ON-TARGETplus Non-targeting smart pool siRNAs (D-001810-10-05, Dharmacon) were transfected into cells using Lipofectamine RNAiMAX (Thermo Fisher Scientific, catalog no. 13778150) following the manufacturer's protocol. The final concentration was 30 nM. The cells were collected for assay after transfection of siRNA for 48 h.

Total RNA was extracted from cells or testes (liquid nitrogen treatment) using TRIzol (Takara). Total RNA (2 μg) was reverse transcribed in a total volume of 20 μL, including 5× RT SuperMix (Vazyme, China), RNase free ddH_2O and template RNA. Aliquots of the RT products were used for qRT-PCR analysis. Each reaction consisted of 10 μL of SYBR Green (1:60,000 final concentration), 0.8 μL of 40 nM sense and antisense primers, 0.8 μL of cDNA, and 8.36 μL of H_2O, to a total volume of 20 μL. Each experiment was performed in duplicate and was repeated three times. RT-PCR used SYBR Green (Bio-Rad) or the Mx3005P-qRT-PCR system (Stratagene). The gene-specific primers were as follows: REGγ sense primer: 5-ACA AGTGAGGCAGAAGAC-3; REGγ antisense primer: 5-ATCATGGCTATTGGTGAG-3; PLZF sense primer: 5-TCAATGCGGTGCCCAGTTCTCA-3; PLZF antisense primer: 5-AGTGCGCTTTGTGCCTGAAAGC-3; β-actin sense primer: 5-CGTCATACTCCTGCTTGCTG-3; β-actin antisense primer: 5-GTACGCCAACACAGTGCTG-3.

Western Blotting Analysis

Protein samples were prepared using radioimmunoprecipitation assay (RIPA) lysis buffer (50 mM Tris-HCl [pH 7.5], 150 mM NaCl, 1% sodium deoxycholate, 1% Triton X-100, 0.1% SDS, 5 mM EDTA, 1 mM Na_3VO_4, 5–10 mM NaF) and western blot analysis of proteins extracted from cells was performed as described previously (Li et al., 2007; Liu et al., 2017). Equivalent amounts of total protein were separated in a 10% SDS-PAGE gel, and immunoblots were analyzed using primary antibodies specific for REGγ, p21, p53, PLZF, MVH, SCP3, and β-actin (1:1,000 dilution) overnight. After incubation with a fluorescent-labeled secondary antibody (1:5,000 dilution), specific signals for proteins were visualized by an LI-COR Odyssey Infrared Imaging System.

Luciferase Assay

Cells were transfected with pGL3 luciferase PLZF, pGL3 luciferase PLZF deletion 583-556, or the pGL3-Basic vector and harvested after 36 h. The cells were washed with cold PBS three times after transfection for 24 h, then lysed in the lysis buffer provided with the Luciferase Assay Kit (Promega). After one cycle of freezing and thawing, the cell lysates were collected and centrifuged at 4°C at 12,000 × *g* for 10 min. The supernatant was then collected, and 20 μL was added to an equal amount of luciferase assay substrate, twice for each lysate. Luminescence was measured as relative light units, and LUMIstar OPTIMA (BMG LABTECH) was used to take the reading of the luciferase assay. The primers

to constructed pGL3 luciferase PLZF and pGL3 luciferase PLZF deletion 583-556 are as follows:

5′-CTAGCTAGCTTTTGTGCATCCTTTTCTCCCC-3′; 5′-CCGCT CGAGGATGCTCCCCTGGGCTCAG-3′;

5′-CAGATCTGGGACCACTGGTTGTCTCCTAAG-3′;

5′-CTTAGGAGACAACCAGTGGTCCCAGATCTG-3′.

Each assay was repeated at least three times. Fold expression values were represented as the mean of the three experiments (Li et al., 2015).

ChIP Assay

ChIP experiments were performed as described previously (Li et al., 2015). Testes were lysed in RIPA lysis buffer. The lysates were then sonicated to result in DNA fragments of 200–1,000 bp in length. Cellular debris was removed by centrifugation and the lysates were diluted 1:10 in ChIP dilution buffer (0.01% SDS, 1.1% Triton X-100, 1.2 mM EDTA, 16.7 mM NaCl, protease inhibitors, and 16.7 mM Tris-HCl [pH 8.1]). The samples were immunoprecipitated with indicated antibodies (immunoglobulin G [IgG], p53) overnight. DNA-protein immunocomplexes were isolated with protein A agarose beads for 2 h. The beads were washed, eluted in 250 mL elution buffer (1% SDS and 100 mM $NaHCO_3$), and crosslinking was reversed by adding NaCl to a final concentration of 200 mM and incubating overnight. The DNA was recovered by phenol/chloroform/isoamyl alcohol (25/24/1) extractions and precipitated with 0.1 volume of 3 M sodium acetate (pH 5.2) and 2 volumes of ethanol using glycogen as a carrier. PCR amplification of the genomic fragments was performed with specific primers flanking putative binding sites on the PLZF promoter. The PCR products were electrophoresed in agarose gels and visualized by ethidium bromide. ChIP primer sequences are as follows: forward: 5-CTTAAGCGTGCAAGGACAGA-3; reverse: 5-TAAAAG TAAGAAGCATTCGGCT-3.

Immunohistochemistry, Immunofluorescence, and TUNEL Analyses

Testes were fixed in Bouin's solution overnight at room temperature and were then dehydrated through a graded series of ethanol and embedded in paraffin. For IHC analysis, slides were boiled in Buffer TE (10 mM Tris, 1 mM EDTA [pH 9.0]) for 20 min. After washing with PBS 3 times, the sections were permeated in H_2O_2 for 10 min, blocked with 5% BSA in PBS for 10 min at room temperature, and incubated overnight at 4°C with the primary antibodies at different concentrations. Subsequently, following three washes with PBS, the slides were incubated for 1 h with biotinylated goat anti-rabbit antibody IgG and then for 30 min with streptavidin-horseradish peroxidase peroxidase. The color reaction product was visualized by using diaminobenzidine-H_2O_2 as a substrate for peroxidase. All sections were counterstained with hematoxylin. For the immunofluorescence staining, dewax of the slides was performed as for the IHC steps. The rest of the steps were performed as described previously (Liu et al., 2010, 2015). To generate the percent of SCP3 cells, $SCP3^+$ cell counts were divided by $PLZF^+$ cells based on the positive staining per view. For TUNEL analysis, slides were permeabilized with 10 mg/mL proteinase K (prepared with 10 mM Tris/HCl, [pH 7.4] buffer) for 30 min. For the experimental group, a TUNEL reaction solution of 50 μL TdT and 450 μL of the fluorescein-labeled dUTP solution was used; the negative control group was incubated with 500 μL of fluorescein-labeled dUTP solution only. After rinsing, 50 μL of the TUNEL reaction mixture was added to the experimental tissue samples (for the negative control group only about 50 μL of dUTP solution was added). The sections were placed in a wet box at 37°C for 1 h in the dark. Sections were counterstained with DAPI for 3–5 min and mounted in media (VECTASHIELD Antifade Mounting Mmedium, Vector Laboratories, Burlingame, CA) and coverslips.

Statistics

Quantitative data were displayed as mean ± SEM or SD of independent samples using Prism software (GraphPad software). Statistical analysis of values was performed using two-tailed Student's t test.

SUPPLEMENTAL INFORMATION

Supplemental Information can be found online at https://doi.org/10.1016/j.stemcr.2019.07.010.

AUTHOR CONTRIBUTIONS

L.L., X.G., H.C., C.C., D.Z., L.G., S.S., Q.W., and X.M. conducted the experiments. L.L., H.C., and J.L. prepared the figures. L.L., Y.X., and J.L. analyzed the data. L.L., X.L., and J.L. planned the project and wrote the manuscript with the help of T.M.C. and B.Z. T.M.C. and B.D. edited the manuscript. L.L., Y.X., and X.L. supervised the project.

ACKNOWLEDGMENTS

This work was supported by the National Basic Research Program of China (2016YFC0902102 and 2015CB910402). This study was also funded by the National Basic Research Program (2011CB504200 and 2015CB910403). This work was also supported in part by grants from the National Natural Science Foundation of China (81401837, 81671446, 81471066, 81261120555, 31200878, 31071875, 81271742, 31401012, and 31730017), the Science and Technology Commission of Shanghai Municipality (19140900400, 14430712100), the Shanghai Rising-Star Program (16QA1401500), and the Shanghai Natural Science Foundation (17ZR1407900, 16ZR1410000, 12ZR1409300, and 14ZR1411400).

Received: April 10, 2018
Revised: July 10, 2019
Accepted: July 15, 2019
Published: August 8, 2019

REFERENCES

Ali, A., Wang, Z., Fu, J., Ji, L., Liu, J., Li, L., Wang, H., Chen, J., Caulin, C., Myers, J.N., et al. (2013). Differential regulation of the REGgamma-proteasome pathway by p53/TGF-beta signalling and mutant p53 in cancer cells. Nat. Commun. *4*, 2667.

Barton, L.F., Runnels, H.A., Schell, T.D., Cho, Y., Gibbons, R., Tevethia, S.S., Deepe, G.S., Jr., and Monaco, J.J. (2004). Immune defects in 28-kDa proteasome activator gamma-deficient mice. J. Immunol. *172*, 3948–3954.

Baum, J.S., St George, J.P., and McCall, K. (2005). Programmed cell death in the germline. Semin. Cell Dev. Biol. *16*, 245–259.

Beumer, T.L., Roepers-Gajadien, H.L., Gademan, I.S., van Buul, P.P., Gil-Gomez, G., Rutgers, D.H., and de Rooij, D.G. (1998). The role of the tumor suppressor p53 in spermatogenesis. Cell Death Differ. *5*, 669–677.

Buaas, F.W., Kirsh, A.L., Sharma, M., McLean, D.J., Morris, J.L., Griswold, M.D., de Rooij, D.G., and Braun, R.E. (2004). Plzf is required in adult male germ cells for stem cell self-renewal. Nat. Genet. *36*, 647–652.

Chen, X., Barton, L.F., Chi, Y., Clurman, B.E., and Roberts, J.M. (2007). Ubiquitin-independent degradation of cell-cycle inhibitors by the REGgamma proteasome. Mol. Cell *26*, 843–852.

Chen, D., Zheng, W., Lin, A., Uyhazi, K., Zhao, H., and Lin, H. (2012). Pumilio 1 suppresses multiple activators of p53 to safeguard spermatogenesis. Curr. Biol. *22*, 420–425.

Chen, H., Gao, X., Sun, Z., Wang, Q., Zuo, D., Pan, L., Li, K., Chen, J., Chen, G., Hu, K., et al. (2017a). REGgamma accelerates melanoma formation by regulating Wnt/beta-catenin signalling pathway. Exp. Dermatol. *26*, 1118–1124.

Chen, S., Wang, L., Yao, X., Chen, H., Xu, C., Tong, L., Shah, A., Huang, T., Chen, G., Chen, J., et al. (2017b). miR-195-5p is critical in REGgamma-mediated regulation of wnt/beta-catenin pathway in renal cell carcinoma. Oncotarget *8*, 63986–64000.

Choi, W.I., Kim, M.Y., Jeon, B.N., Koh, D.I., Yun, C.O., Li, Y., Lee, C.E., Oh, J., Kim, K., and Hur, M.W. (2014). Role of promyelocytic leukemia zinc finger (PLZF) in cell proliferation and cyclin-dependent kinase inhibitor 1A (p21WAF/CDKN1A) gene repression. J. Biol. Chem. *289*, 18625–18640.

Costoya, J.A., Hobbs, R.M., Barna, M., Cattoretti, G., Manova, K., Sukhwani, M., Orwig, K.E., Wolgemuth, D.J., and Pandolfi, P.P. (2004). Essential role of Plzf in maintenance of spermatogonial stem cells. Nat. Genet. *36*, 653–659.

Costoya, J.A., Hobbs, R.M., and Pandolfi, P.P. (2008). Cyclin-dependent kinase antagonizes promyelocytic leukemia zinc-finger through phosphorylation. Oncogene *27*, 3789–3796.

Dong, S., Jia, C., Zhang, S., Fan, G., Li, Y., Shan, P., Sun, L., Xiao, W., Li, L., Zheng, Y., et al. (2013). The REGgamma proteasome regulates hepatic lipid metabolism through inhibition of autophagy. Cell Metab. *18*, 380–391.

el-Deiry, W.S., Kern, S.E., Pietenpol, J.A., Kinzler, K.W., and Vogelstein, B. (1992). Definition of a consensus binding site for p53. Nat. Genet. *1*, 45–49.

Falender, A.E., Freiman, R.N., Geles, K.G., Lo, K.C., Hwang, K., Lamb, D.J., Morris, P.L., Tjian, R., and Richards, J.S. (2005). Maintenance of spermatogenesis requires TAF4b, a gonad-specific subunit of TFIID. Genes Dev. *19*, 794–803.

Filipponi, D., Hobbs, R.M., Ottolenghi, S., Rossi, P., Jannini, E.A., Pandolfi, P.P., and Dolci, S. (2007). Repression of kit expression by Plzf in germ cells. Mol. Cell. Biol. *27*, 6770–6781.

Flores, E.R., Tsai, K.Y., Crowley, D., Sengupta, S., Yang, A., McKeon, F., and Jacks, T. (2002). p63 and p73 are required for p53-dependent apoptosis in response to DNA damage. Nature *416*, 560–564.

Fridman, J.S., and Lowe, S.W. (2003). Control of apoptosis by p53. Oncogene *22*, 9030–9040.

Fujisawa, M., Shirakawa, T., Fujioka, H., Gotoh, A., Okada, H., Arakawa, S., and Kamidono, S. (2001). Adenovirus-mediated p53 gene transfer to rat testis impairs spermatogenesis. Arch. Androl. *46*, 223–231.

Helsel, A.R., Yang, Q.E., Oatley, M.J., Lord, T., Sablitzky, F., and Oatley, J.M. (2017). ID4 levels dictate the stem cell state in mouse spermatogonia. Development *144*, 624–634.

Hobbs, R.M., Seandel, M., Falciatori, I., Rafii, S., and Pandolfi, P.P. (2010). Plzf regulates germline progenitor self-renewal by opposing mTORC1. Cell *142*, 468–479.

Hofmann, M.C. (2008). Gdnf signaling pathways within the mammalian spermatogonial stem cell niche. Mol. Cell. Endocrinol. *288*, 95–103.

Huang, L., Haratake, K., Miyahara, H., and Chiba, T. (2016). Proteasome activators, PA28gamma and PA200, play indispensable roles in male fertility. Sci. Rep. *6*, 23171.

Kachnic, L.A., Wu, B., Wunsch, H., Mekeel, K.L., DeFrank, J.S., Tang, W., and Powell, S.N. (1999). The ability of p53 to activate downstream genes p21(WAF1/cip1) and MDM2, and cell cycle arrest following DNA damage is delayed and attenuated in scid cells deficient in the DNA-dependent protein kinase. J. Biol. Chem. *274*, 13111–13117.

Khor, B., Bredemeyer, A.L., Huang, C.Y., Turnbull, I.R., Evans, R., Maggi, L.B., Jr., White, J.M., Walker, L.M., Carnes, K., Hess, R.A., et al. (2006). Proteasome activator PA200 is required for normal spermatogenesis. Mol. Cell. Biol. *26*, 2999–3007.

Kubota, H., Avarbock, M.R., and Brinster, R.L. (2004). Growth factors essential for self-renewal and expansion of mouse spermatogonial stem cells. Proc. Natl. Acad. Sci. U S A *101*, 16489–16494.

La, H.M., Makela, J.A., Chan, A.L., Rossello, F.J., Nefzger, C.M., Legrand, J.M.D., De Seram, M., Polo, J.M., and Hobbs, R.M. (2018). Identification of dynamic undifferentiated cell states within the male germline. Nat. Commun. *9*, 2819.

Li, J.Y., English, M.A., Ball, H.J., Yeyati, P.L., Waxman, S., and Licht, J.D. (1997). Sequence-specific DNA binding and transcriptional regulation by the promyelocytic leukemia zinc finger protein. J. Biol. Chem. *272*, 22447–22455.

Li, X., Lonard, D.M., Jung, S.Y., Malovannaya, A., Feng, Q., Qin, J., Tsai, S.Y., Tsai, M.J., and O'Malley, B.W. (2006). The SRC-3/AIB1 coactivator is degraded in a ubiquitin- and ATP-independent manner by the REGgamma proteasome. Cell *124*, 381–392.

Li, X., Amazit, L., Long, W., Lonard, D.M., Monaco, J.J., and O'Malley, B.W. (2007). Ubiquitin- and ATP-independent proteolytic turnover of p21 by the REGgamma-proteasome pathway. Mol. Cell *26*, 831–842.

Li, L., Zhao, D., Wei, H., Yao, L., Dang, Y., Amjad, A., Xu, J., Liu, J., Guo, L., Li, D., et al. (2013). REGgamma deficiency promotes premature aging via the casein kinase 1 pathway. Proc. Natl. Acad. Sci. U S A *110*, 11005–11010.

Li, L., Dang, Y., Zhang, J., Yan, W., Zhai, W., Chen, H., Li, K., Tong, L., Gao, X., Amjad, A., et al. (2015). REGgamma is critical for skin carcinogenesis by modulating the Wnt/beta-catenin pathway. Nat. Commun. *6*, 6875.

Liu, J., Yu, G., Zhao, Y., Zhao, D., Wang, Y., Wang, L., Li, L., Zeng, Y., Dang, Y., Wang, C., et al. (2010). REGgamma modulates p53

activity by regulating its cellular localization. J. Cell Sci. *123*, 4076–4084.

Liu, J., Cho, S.N., Akkanti, B., Jin, N., Mao, J., Long, W., Chen, T., Zhang, Y., Tang, X., Wistub, I.I., et al. (2015). ErbB2 pathway activation upon Smad4 loss promotes lung tumor growth and metastasis. Cell Rep. *10*, 1599–1613.

Liu, J., Cho, S.N., Wu, S.P., Jin, N., Moghaddam, S.J., Gilbert, J.L., Wistuba, I., and DeMayo, F.J. (2017). Mig-6 deficiency cooperates with oncogenic Kras to promote mouse lung tumorigenesis. Lung Cancer *112*, 47–56.

Lovasco, L.A., Gustafson, E.A., Seymour, K.A., de Rooij, D.G., and Freiman, R.N. (2015). TAF4b is required for mouse spermatogonial stem cell development. Stem Cells *33*, 1267–1276.

Lv, Y., Meng, B., Dong, H., Jing, T., Wu, N., Yang, Y., Huang, L., Moses, R.E., O'Malley, B.W., Mei, B., et al. (2016). Upregulation of GSK3beta contributes to brain disorders in elderly REGgamma-knockout mice. Neuropsychopharmacology *41*, 1340–1349.

Marcet-Ortega, M., Pacheco, S., Martinez-Marchal, A., Castillo, H., Flores, E., Jasin, M., Keeney, S., and Roig, I. (2017). p53 and TAp63 participate in the recombination-dependent pachytene arrest in mouse spermatocytes. PLoS Genet. *13*, e1006845.

Matson, C.K., Murphy, M.W., Sarver, A.L., Griswold, M.D., Bardwell, V.J., and Zarkower, D. (2011). DMRT1 prevents female reprogramming in the postnatal mammalian testis. Nature *476*, 101–104.

Matsumoto, A.M., and Bremner, W.J. (1987). Endocrinology of the hypothalamic-pituitary-testicular axis with particular reference to the hormonal control of spermatogenesis. Baillieres Clin. Endocrinol. Metab. *1*, 71–87.

Menendez, D., Inga, A., and Resnick, M.A. (2009). The expanding universe of p53 targets. Nat. Rev. Cancer *9*, 724–737.

Meng, X., Lindahl, M., Hyvonen, M.E., Parvinen, M., de Rooij, D.G., Hess, M.W., Raatikainen-Ahokas, A., Sainio, K., Rauvala, H., Lakso, M., et al. (2000). Regulation of cell fate decision of undifferentiated spermatogonia by GDNF. Science *287*, 1489–1493.

Oatley, J.M., Avarbock, M.R., Telaranta, A.I., Fearon, D.T., and Brinster, R.L. (2006). Identifying genes important for spermatogonial stem cell self-renewal and survival. Proc. Natl. Acad. Sci. U S A *103*, 9524–9529.

Oren, M. (1994). Relationship of p53 to the control of apoptotic cell death. Semin. Cancer Biol. *5*, 221–227.

Roos, W.P., and Kaina, B. (2006). DNA damage-induced cell death by apoptosis. Trends Mol. Med. *12*, 440–450.

Schwartz, D., Goldfinger, N., Kam, Z., and Rotter, V. (1999). p53 controls low DNA damage-dependent premeiotic checkpoint and facilitates DNA repair during spermatogenesis. Cell Growth Differ. *10*, 665–675.

Takubo, K., Ohmura, M., Azuma, M., Nagamatsu, G., Yamada, W., Arai, F., Hirao, A., and Suda, T. (2008). Stem cell defects in ATM-deficient undifferentiated spermatogonia through DNA damage-induced cell-cycle arrest. Cell Stem Cell *2*, 170–182.

Walker, W.H. (2009). Molecular mechanisms of testosterone action in spermatogenesis. Steroids *74*, 602–607.

Wang, H., Zhao, R., Guo, C., Jiang, S., Yang, J., Xu, Y., Liu, Y., Fan, L., Xiong, W., Ma, J., et al. (2016). Knockout of BRD7 results in impaired spermatogenesis and male infertility. Sci. Rep. *6*, 21776.

Xu, J., Zhou, L., Ji, L., Chen, F., Fortmann, K., Zhang, K., Liu, Q., Li, K., Wang, W., Wang, H., et al. (2016). The REGgamma-proteasome forms a regulatory circuit with IkappaBvarepsilon and NFkappaB in experimental colitis. Nat. Commun. *7*, 10761.

Xu, L., Lu, Y., Han, D., Yao, R., Wang, H., Zhong, S., Luo, Y., Han, R., Li, K., Fu, J., et al. (2017). Rnf138 deficiency promotes apoptosis of spermatogonia in juvenile male mice. Cell Death Dis. *8*, e2795.

Yu, G., Zhao, Y., He, J., Lonard, D.M., Mao, C.A., Wang, G., Li, M., and Li, X. (2010). Comparative analysis of REG{gamma} expression in mouse and human tissues. J. Mol. Cell Biol. *2*, 192–198.

Zhang, Z., and Zhang, R. (2008). Proteasome activator PA28 gamma regulates p53 by enhancing its MDM2-mediated degradation. EMBO J. *27*, 852–864.

Zhang, T., Oatley, J., Bardwell, V.J., and Zarkower, D. (2016). DMRT1 is required for mouse spermatogonial stem cell maintenance and replenishment. PLoS Genet. *12*, e1006293.

Zhou, Q., Wang, M., Yuan, Y., Wang, X., Fu, R., Wan, H., Xie, M., Liu, M., Guo, X., Zheng, Y., et al. (2016). Complete meiosis from embryonic stem cell-derived germ cells in vitro. Cell Stem Cell *18*, 330–340.

Zhu, H., and Gooderham, N. (2002). Neoplastic transformation of human lung fibroblast MRC-5 SV2 cells induced by benzo[a]pyrene and confluence culture. Cancer Res. *62*, 4605–4609.

Zuo, Q., Cheng, S., Huang, W., Bhatti, M.Z., Xue, Y., Zhang, Y., Zhang, B., Li, L., Wu, L., Fu, J., et al. (2017). REGgamma contributes to regulation of hemoglobin and hemoglobin delta subunit. Oxid Med. Cell. Longev. *2017*, 7295319.

Stem Cell Reports, Volume *13*

Supplemental Information

The REGγ-Proteasome Regulates Spermatogenesis Partially by P53-PLZF Signaling

Xiao Gao, Hui Chen, Jian Liu, Shihui Shen, Qingwei Wang, Tracy M. Clement, Brian J. Deskin, Caiyu Chen, Dengpan Zhao, Lu Wang, Linjie Guo, Xueqing Ma, Bianhong Zhang, Yunfei Xu, Xiaotao Li, and Lei Li

Supplemental Figure Legends

Figure S1. Expression of REGγ In Male Mouse Germ Cells. Related to Figure 1.

(A) Immunofluorescence (IF) staining of REGγ in p7, p10 and 2m mouse testes. (Scale bar, 50μm).

(B) The number of pups in breeding trial experiments.

(C) Photograph of mouse testes.

(D) Quantification of mouse whole weight. Error bars represent SEM.

(E) H&E staining of mouse testes. (Scale bar, 50μm).

Figure S2. Decreased Proportion of Undifferentiated Spermatogonia. Related to Figure 3.

(A) IF staining of PLZF and REGγ in control and REGγ$^{-/-}$ testes at p10. The DNA was stained with DAPI. (Scale bar, 50μm).

(B-C) RT-qPCR analysis of marker gene expression in testes at p7 from REGγ$^{+/+}$ and REGγ$^{-/-}$ mouse testes, with actin as an internal control. (n = 3, ** $P < 0.01$, *** $P < 0.001$). Error bars represent SEM.

Figure S3. REGγ$^{-/-}$ testes induced p53 expression and apoptosis while reduce PLZF expression. Related to Figure 5.

(A-B) IF staining analysis of p53, REGγ and PLZF in REGγ$^{+/+}$ and REGγ$^{-/-}$ testes. Upper panel: IF staining at p10 (A) and 2m (B), (Scale bar, 50μm).

(C) Western Blotting analysIs of cleaved PARP, PARP, REGγ and β-actin in adult

REGγ$^{+/+}$ and REGγ$^{-/-}$ male testes treated Cisplatin in different time. β-actin was used as a loading control.

(D) Detection of apoptotic cells and SCP3 double staining in the testes of REGγ$^{-/-}$ mice at 2 months of age. (Scale bar, 50μm).

Figure S4. Genetic attenuation of p53 restores spermatogenesis in REGγ $^{-/-}$ mice. Related to Figure 6. (A) Histological appearances of testes at p10 from p53$^{+/-}$REGγ$^{-/-}$ and p53$^{+/+}$REGγ$^{-/-}$ male mice. (Scale bar, 50μm).

Figure S1

A

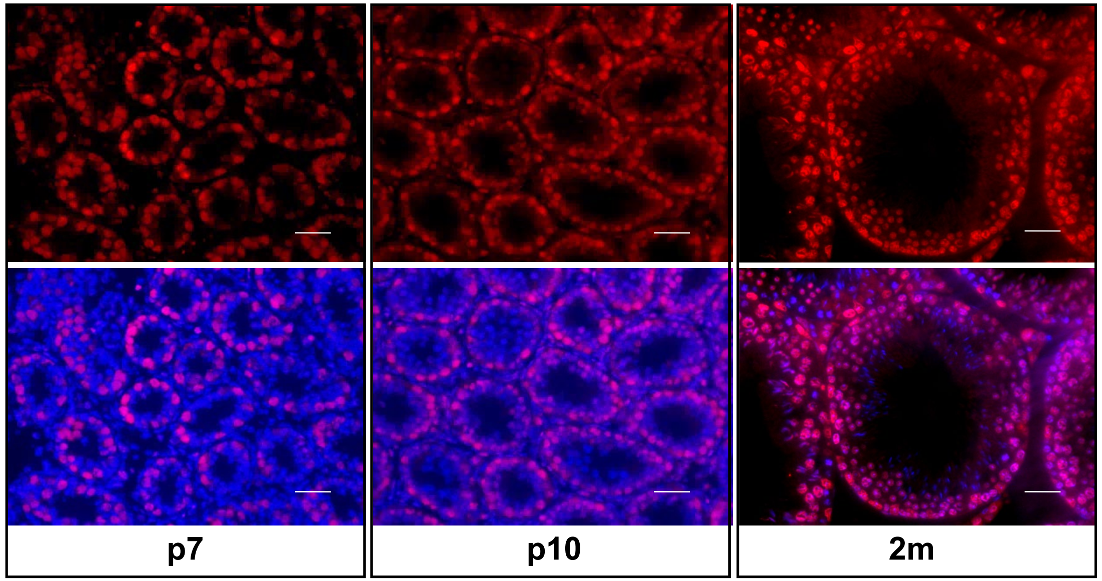

B

The detail information of breeding trials of Figure 1B.

Breeding Cage	Pups (1st litter)	Pups (2nd litter)	Pups (3rd litter)	Pups (4th litter)
REGγ+/+ (♂) x REGγ+/+ (♀)	8	6	9	12
REGγ+/+ (♂) x REGγ+/+ (♀)	5	12	9	6
REGγ+/+ (♂) x REGγ+/+ (♀)	4	9	11	10
REGγ+/+ (♂) x REGγ+/+ (♀)	8	11	9	12
REGγ+/+ (♂) x REGγ+/+ (♀)	7	10	9	13
Total number of pups	180			
Average number of pups per litter	9			
Breeding Cage	Pups (1st litter)	Pups (2nd litter)	Pups (3rd litter)	Pups (4th litter)
REGγ-/-(♂) x REGγ+/+ (♀)	4	5	4	3
REGγ-/-(♂) x REGγ+/+ (♀)	6	6	5	6
REGγ-/-(♂) x REGγ+/+ (♀)	2	4	5	4
REGγ-/-(♂) x REGγ+/+ (♀)	4	8	3	4
REGγ-/-(♂) x REGγ+/+ (♀)	3	6	11	4
Total number of pups	97			
Average number of pups per litter	4.85			

C D E

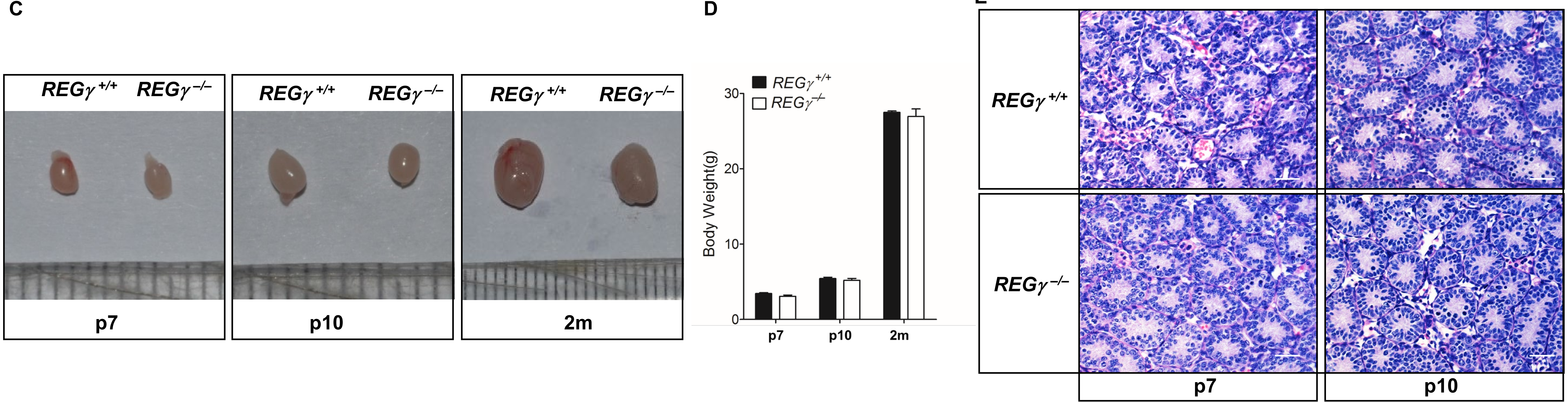

Figure S2

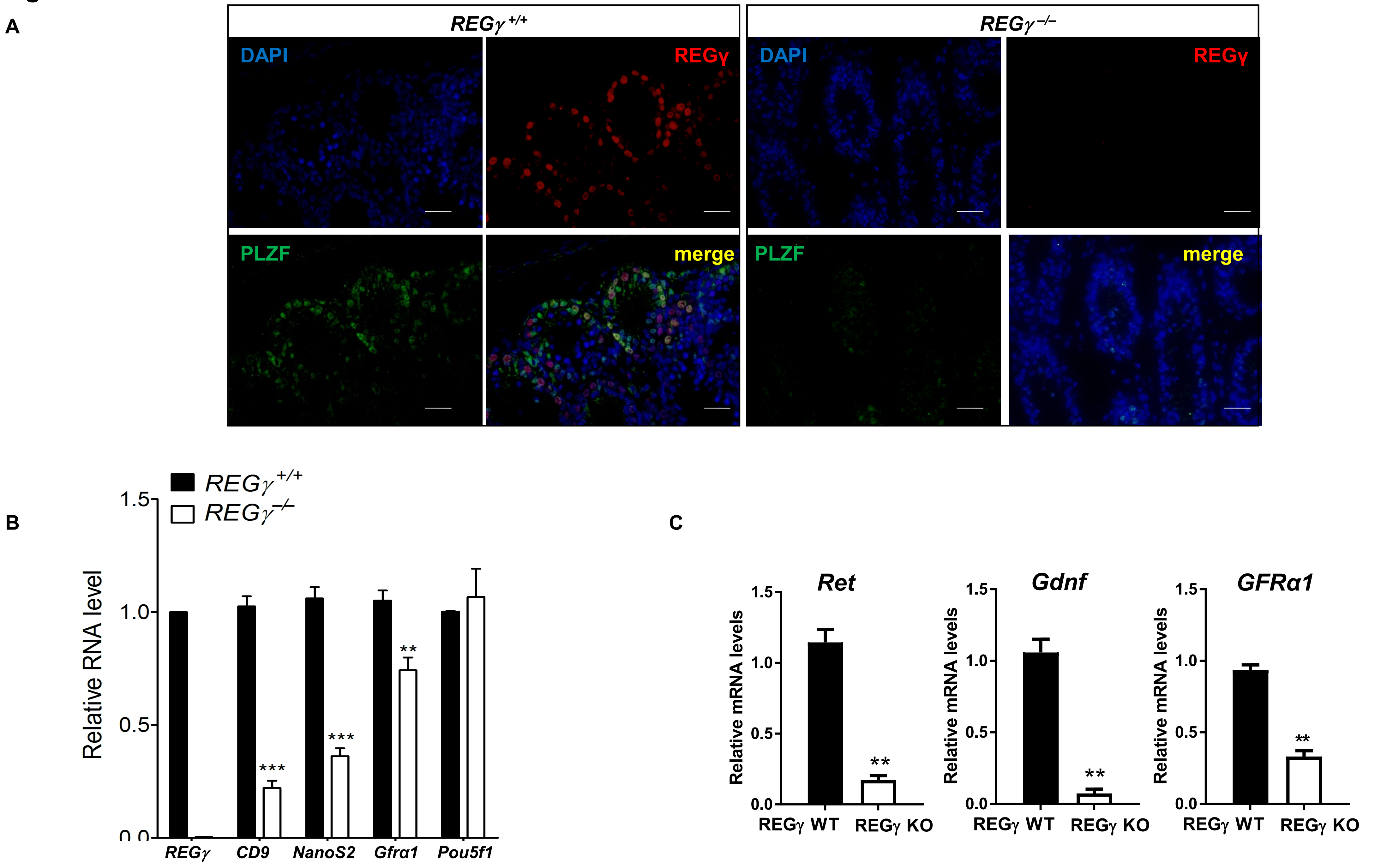

A
REGγ +/+
REGγ −/−
DAPI
REGγ
PLZF
merge
B
REGγ +/+
REGγ −/−
Relative RNA level
1.5
1.0
0.5
0.0
***
***
**
REGγ
CD9
NanoS2
Gfrα1
Pou5f1
C
Ret
Gdnf
GFRα1
Relative mRNA levels
1.5
1.0
0.5
0.0
**
**
**
REGγ WT
REGγ KO

Figure S3

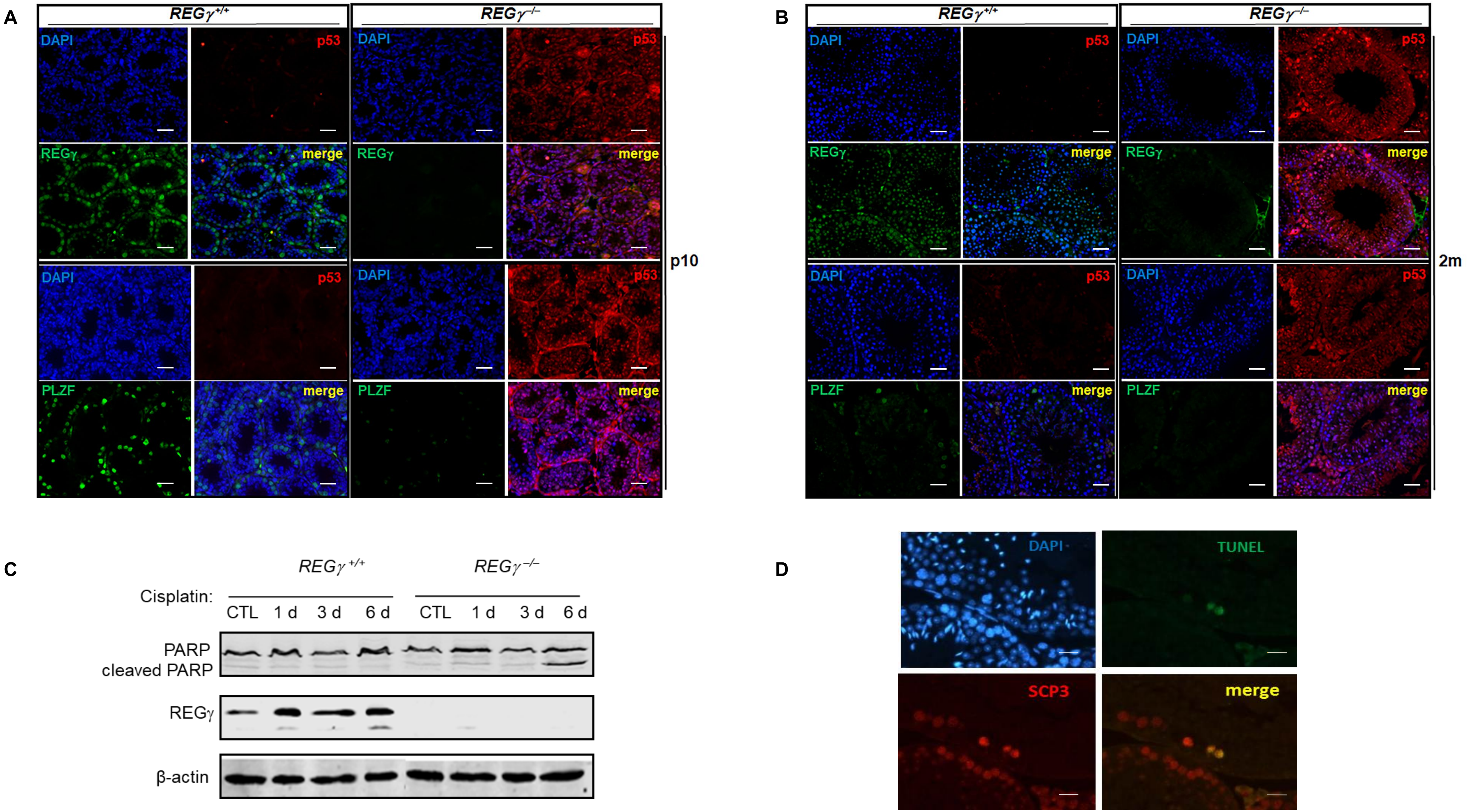

A
REGγ+/+
REGγ−/−
DAPI
p53
REGγ
merge
PLZF
p10
B
2m
C
REGγ +/+
REGγ −/−
Cisplatin:
CTL
1 d
3 d
6 d
PARP
cleaved PARP
REGγ
β-actin
D
DAPI
TUNEL
SCP3
merge

Figure S4

A

p53$^{+/-}$REG$\gamma^{-/-}$

p53$^{+/+}$REG$\gamma^{-/-}$

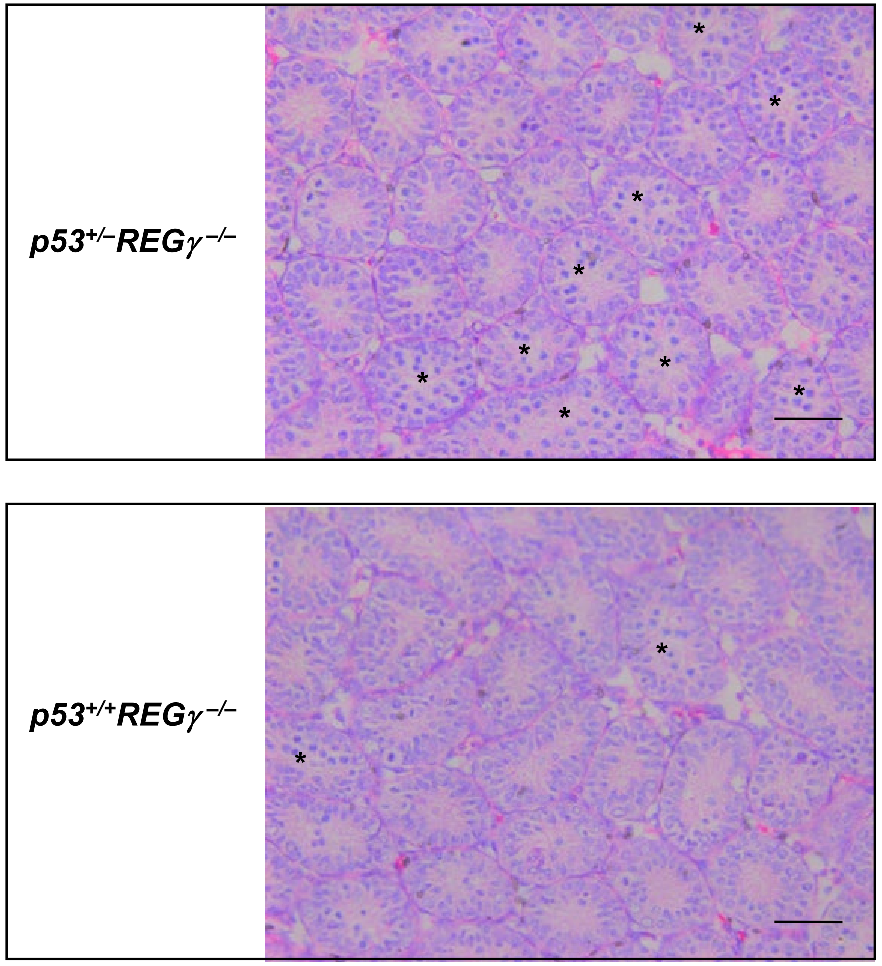

Supplement: Document S2. Article plus Supplemental Information [file mmc2.pdf]
